# The Amidation Step of Diphthamide Biosynthesis in Yeast Requires *DPH6*, a Gene Identified through Mining the *DPH1*-*DPH5* Interaction Network

**DOI:** 10.1371/journal.pgen.1003334

**Published:** 2013-02-28

**Authors:** Shanow Uthman, Christian Bär, Viktor Scheidt, Shihui Liu, Sara ten Have, Flaviano Giorgini, Michael J. R. Stark, Raffael Schaffrath

**Affiliations:** 1 Department of Genetics, University of Leicester, Leicester, United Kingdom; 2 Institut für Biologie, FG Mikrobiologie, Universität Kassel, Kassel, Germany; 3 Laboratory of Parasitic Diseases, National Institute of Allergy and Infectious Diseases, National Institutes of Health, Bethesda, Maryland, United States of America; 4 Centre for Gene Regulation and Expression, College of Life Sciences, MSI/WTB Complex, University of Dundee, Dundee, Scotland; Aarhus University, Denmark

## Abstract

Diphthamide is a highly modified histidine residue in eukaryal translation elongation factor 2 (eEF2) that is the target for irreversible ADP ribosylation by diphtheria toxin (DT). In *Saccharomyces cerevisiae*, the initial steps of diphthamide biosynthesis are well characterized and require the *DPH1-DPH5* genes. However, the last pathway step—amidation of the intermediate diphthine to diphthamide—is ill-defined. Here we mine the genetic interaction landscapes of *DPH1-DPH5* to identify a candidate gene for the elusive amidase (*YLR143w*/*DPH6*) and confirm involvement of a second gene (*YBR246w*/*DPH7*) in the amidation step. Like *dph1-dph5*, *dph6* and *dph7* mutants maintain eEF2 forms that evade inhibition by DT and sordarin, a diphthamide-dependent antifungal. Moreover, mass spectrometry shows that *dph6* and *dph7* mutants specifically accumulate diphthine-modified eEF2, demonstrating failure to complete the final amidation step. Consistent with an expected requirement for ATP in diphthine amidation, Dph6 contains an essential adenine nucleotide hydrolase domain and binds to eEF2. Dph6 is therefore a candidate for the elusive amidase, while Dph7 apparently couples diphthine synthase (Dph5) to diphthine amidation. The latter conclusion is based on our observation that *dph7* mutants show drastically upregulated interaction between Dph5 and eEF2, indicating that their association is kept in check by Dph7. Physiologically, completion of diphthamide synthesis is required for optimal translational accuracy and cell growth, as indicated by shared traits among the *dph* mutants including increased ribosomal −1 frameshifting and altered responses to translation inhibitors. Through identification of Dph6 and Dph7 as components required for the amidation step of the diphthamide pathway, our work paves the way for a detailed mechanistic understanding of diphthamide formation.

## Author Summary

Diphthamide is an unusual modified amino acid found uniquely in a single protein, eEF2, which is required for cells to synthesize new proteins. The name refers to its target function for eEF2 inactivation by diphtheria toxin, the disease-inducing agent produced by the pathogen *Corynebacterium diphtheriae*. Why cells require eEF2 to contain diphthamide is unclear, although mice unable to make it fail to complete embryogenesis. Cells generate diphthamide by modifying a specific histidine residue in eEF2 using a three-step biosynthetic pathway, the first two steps of which are well defined. However, the enzyme(s) involved in the final amidation step are unknown. Here we integrate genomic and molecular approaches to identify a candidate for the elusive amidase (Dph6) and confirm involvement of a second protein (Dph7) in the amidation step, showing that failure to synthesize diphthamide affects the accuracy of protein synthesis. In contrast to Dph6, however, Dph7 may be regulatory. Our data strongly suggest that it promotes dissociation of eEF2 from diphthine synthase (Dph5), which carries out the second step of diphthamide synthesis, and that Dph5 has a novel role as an eEF2 inhibitor when diphthamide synthesis is incomplete.

## Introduction

**Figure 1:**
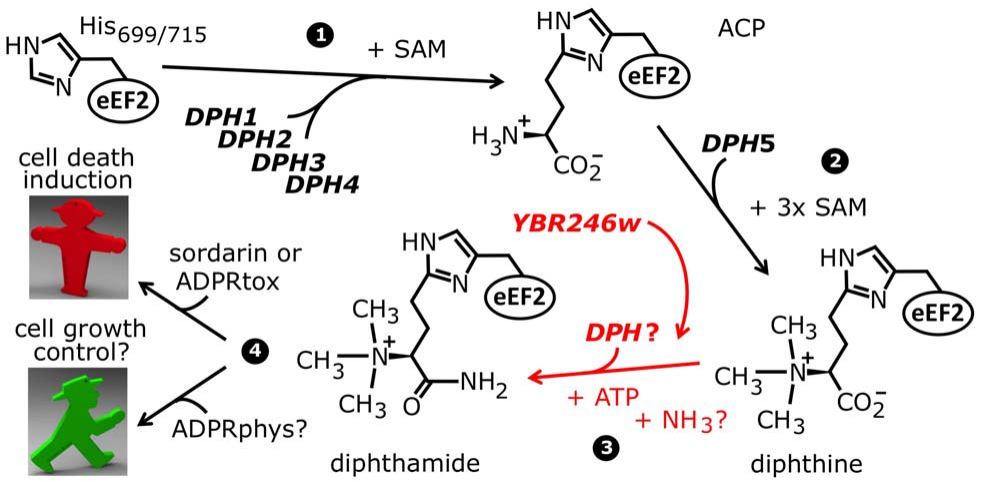
The biosynthetic pathway for modification of eEF2 by diphthamide. For roles played by the *bona fide* diphthamide genes *DPH1–DPH5* in steps 1 and 2 of the pathway, see main text. The ill-defined step 3, conversion of diphthine to diphthamide by amidation, is highlighted (red label). It likely involves ATP and ammonium cofactors in a reaction catalyzed by unidentified *DPH* gene product(s). Step 4 indicates diphthamide can be hijacked for eEF2 inactivation and cell death induction by antifungals, i.e. sordarin and bacterial ADP ribosylase toxins (ADPRtox); alternatively, it has been reported to undergo cell growth related physiological ADP ribosylation (ADPRphys?) by elusive cellular modifier(s). ACP, 2-[3-amino-carboxyl-propyl]-histidine; SAM: S-adenosylmethionine.

Regulation of biological processes by posttranslational modification can involve the function, distribution and interaction capabilities of the modified protein [1]–[3]. Though most modification pathways such as phosphorylation and ubiquitin conjugation target many different proteins, some exceptional ones uniquely target just a single polypeptide [4]. One prominent example is diphthamide formation on eukaryal translation elongation factor 2 (eEF2) [5]. Strikingly, this modification is pathobiologically important because it is hijacked for eEF2 inhibition by sordarin fungicides and by diphtheria toxin (DT) produced by pathovarieties of *Corynebacterium diphtheriae* that cause the severe human disease syndrome diphtheria [6]–[8]. Both agents efficiently block protein synthesis by inactivating the essential function of the modified translation factor, via stalling the diphthamide modified form of eEF2 on ribosomes and irreversible ADP ribosylation of eEF2, respectively [9]–[12]. Diphthamide itself is a highly modified histidine residue on eEF2 – 2-[3-carboxyamido-3-(trimethylamino)-propyl]-histidine – which is conserved from yeast (H_699_) to man (H_715_) (Figure 1) [5], [8], [13]. Intriguingly, it is absent from the bacterial eEF2 analog, EF-G, thus conferring immunity on the DT producer.

Among the archaea and eukarya, diphthamide formation involves a conserved biosynthetic pathway, which has been extensively dissected in *Saccharomyces cerevisiae* via isolation of mutant strains that confer resistance to growth inhibition by DT and sordarin. This has led to the identification of the diphthamide synthesis genes *DPH1-DPH5* [7], [12], [14]–[16] (Figure 1). The first step in diphthamide synthesis involves transfer of a 3-amino-3-carboxypropyl (ACP) radical from S-adenosyl-methionine (SAM) to the histidine imidazole ring, generating the ACP modified intermediate of eEF2 [17]–[19]. ACP radical transfer requires the proteins Dph1-Dph4 [16], where Dph1 and Dph2 are paralogous iron-sulfur cluster containing partner proteins that copurify and interact with Dph3, potentially as part of a multimeric complex [6], [20]–[22]. Dph3 and Dph4 are thought to chaperone Dph1-Dph2 by maintaining their iron-sulfur clusters in redox states required for proper ACP radical generation. In line with this, Dph3 and Dph4 have electron carrier activities [23], [24], while Dph3 (also known as Kti11 [25]) additionally partners with Elongator subunit Elp3 [6], [20], an iron-sulfur cluster and radical SAM enzyme with roles in protein and tRNA modifications [26]–[28].

Formation of diphthine, the second pathway intermediate (Figure 1), requires trimethylation of the amino group in ACP and is catalyzed in yeast by diphthine synthase Dph5, using SAM as methyl donor [29]–[31]. Intriguingly, reconstitution of archaeal Dph5 activity has shown that the trimethylamino group formed in diphthine is prone to elimination in vitro [32]. Finally, the carboxyl group of diphthine is amidated by an elusive ATP dependent diphthamide synthetase (Figure 1). Once fully modified, diphthamide can be efficiently targeted by NAD^+^-dependent ADP ribosylase toxins including DT, *Pseudomonas* exotoxin A [33] and *Vibrio* cholix toxin [34]. However, the intermediate diphthine is also a very weak substrate for inhibitory ADP ribosylation [29], [31]. Together with data showing that growth inhibition by sordarin also depends on *DPH1-DPH5* [6], [7], translation factor eEF2 constitutes an ‘Achilles heel’ for yeast, study of which has provided important insight into the pathobiological relevance of posttranslational protein modification [35].

Physiologically, the function of the diphthamide modification is enigmatic. Yeast mutants unable to synthesize diphthamide confer elevated frequency of ribosomal frameshifting [6], [36] but are viable and grow normally [14], although substitution of the modified histidine in eEF2 by other amino acids confers growth defects in some instances [37]. However, loss of diphthamide synthesis leads to delayed development and is embryonic lethal in homozygous *DPH3* knockout mice [38]–[40]. Together with the association of mammalian *DPH1* with tumorigenesis [16], [38] as well as neuronal and embryonic development, this indicates that diphthamide modification plays an important biological role. Whether or not this implies structural or regulatory roles for diphthamide modified eEF2 remains to be seen, but the latter notion is intriguing given the possibility of endogenous cellular ADP ribosylases that target eEF2 [4].

Interestingly, no DT resistant yeast mutants have been identified to date that affect the final amidation step in the pathway, probably because diphthine is targetable, albeit inefficiently, by ADP ribosylation [29], [31]. Thus amidase-deficient mutants may display DT sensitivity in vivo and thereby escape identification in screens for DT resistant yeast mutants.

Indication that additional proteins are involved in diphthamide biosynthesis has come from recent work on WDR85 and its potential yeast ortholog *YBR246w* [41], [42], while our preliminary investigation of the yeast *DPH1* genetic interaction network [13] implicated both *YBR246w* and *YLR143w* as novel proteins potentially involved in the diphthamide pathway. Here we further exploit yeast genome-wide genetic interaction and chemical genomics databases [43], [44] to demonstrate that *YLR143w* (*DPH6*) and *YBR246W* (*DPH7*) cluster tightly with all known members of the diphthamide gene network. We find that *dph6* and *dph7* mutants phenocopy sordarin and DT traits typical of the *bona fide dph1-dph5* mutants, which are defective in the first two steps of diphthamide synthesis. Importantly, we show that *DPH6* and *DPH7* deletions block the final amidation step of the diphthamide pathway, cause diphthine modified forms of eEF2 to accumulate and consequently abolish ADP ribose acceptor activity upon DT treatment. Thus conversion of diphthine to diphthamide depends on Dph6 and Dph7.

## Results

### Yeast Gene Interaction Databases Predict Diphthamide Functions for *YLR143w* (*DPH6*) and *YBR246w* (*DPH7*)

To identify factors involved in the terminal amidation step of the diphthamide modification pathway (Figure 1), we took advantage of synthetic genetic array (SGA) screens, which previously enabled systematic mapping of genetic interactions among yeast deletion collections using high-density arrays of double mutants [45], [46]. SGA analysis provides the set of genetic interactions for a given gene – the genetic interaction profile – and thereby the phenotypic signatures indicative of functions of both known genes and unassigned ORFs [47]. For example, genes with similar interaction profiles are often functionally related in shared biochemical pathways and/or protein complexes [48], [49]. Consistent with this, SGA analysis revealed that the diphthamide gene network members have highly correlated interaction profiles and tightly cluster in the global genetic interaction landscape from yeast [45].

**Figure 2:**
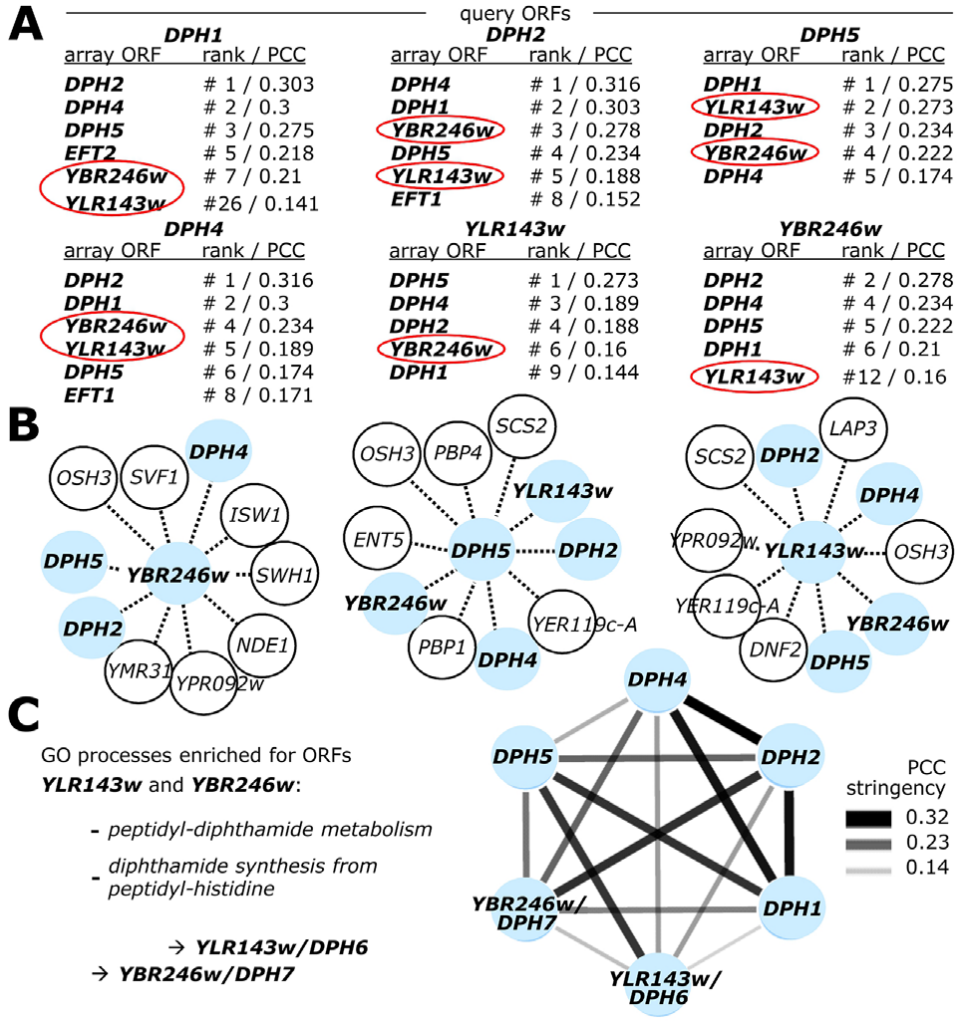
Genome-wide gene interaction databases identify additional diphthamide related candidate genes: *YLR143w*/*DPH6* and *YBR246w*/*DPH7*. (A) SGA database (DRYGIN). Genetic interaction profiles among *DPH1*, *DPH2*, *DPH4*, *DPH5*, *YBR246w* and *YLR143w* query gene deletion strains and 3885 or 4457 array ORF mutants were extracted from data for a total of ∼1700 query strains deposited at DRYGIN (for full details, see excel spread sheet in Table S1). Ranking of top interactors for each query ORF was according to PCC (Pearson correlation coefficient) determination. For simplicity, array ORFs *DPH1*, *DPH2*, *DPH4*, *DPH5*, *EFT1*, *EFT2* (shown in bold) as well as potentially diphthamide related candidate loci *YLR143w* and *YBR246w* (red circles) are listed that score repeatedly as significantly high interactors of the query ORFs. (B) Yeast Fitness database (FitDB). Genes whose deletions phenocluster with the six query ORFs above were extracted from FitDB, which is based on genome-scale co-fitness defect analysis of homozygous yeast deletion mutants in response to greater than 1144 different conditions. For simplicity, the top ten interactors for three of the six query genes (*DPH5*, *YLR143w* and *YBR246w:* pale blue central nodes) above are depicted. (C) Representation of the tightly clustered and expanded *DPH1-DPH7* gene network where nodes (pale blue) correspond to individual *DPH* gene family members and edges connect gene pairs by PCC>0.14. Enhanced gene interaction strength is proportional to PCC stringency. Enriched GO process likelihoods in the diphthamide modification pathway are listed as P-values for the identified candidates *DPH6*/*YLR143w* and *DPH7*/*YBR246w*.

Since our preliminary examination of the yeast genetic interaction landscape placed two uncharacterized yeast ORFs, *YLR143w* and *YBR246w*, within the diphthamide gene network [13], we next examined this network in more detail by mining the SGA DRYGIN database for quantitative *S. cerevisiae* genetic interactions [44], [50]. We compared *DPH1*, *DPH2*, *DPH4*, *DPH5*, *YLR143w* and *YBR246w* gene interactions with every array ORF represented in the SGA network and deposited at DRYGIN, ranking the similarity between all possible pairwise profiles according to their Pearson correlation coefficient (PCC; see Table S1 for full details). As expected, the other known *DPH* genes scored significantly highly among the correlation profiles generated for each specific *DPH* query gene, consistently being ranked among the top ten genetic interactors (Figure 2A). Strikingly, *YLR143w* and *YBR246w* were among the top interactors of *DPH1*, *DPH2*, *DPH4* and *DPH5*, while the most correlated interactors for *YLR143w* and *YBR246w* included each other and several *bone fide DPH* genes (Figure 2A). Such highly correlated interaction patterns suggest that *YLR143w* and *YBR246w* are both functionally interrelated and qualify as candidate ORFs of the pathway for eEF2 modification by diphthamide. In line with this notion, the two eEF2 encoding gene copies, *EFT1* and *EFT2*, also ranked among the top ten interactors of *DPH1*, *DPH2* and *DPH5* (Figure 2A).

For independent validation of these correlations, we searched the FitDB yeast fitness database [51], which contains genome-scale phenotypic profiles for diploid yeast deletion collections in response to more than 1100 different growth conditions [43], [52]. Here, scoring gene interaction profiles by homozygous co-sensitivity revealed that among the top loci to phenocluster with *YBR246w* are *DPH2*, *DPH4* and *DPH5*, while top interactors of *YLR143w* include *DPH4*, *DPH5*, *YBR246w* and *DPH2* (Figure 2B). A similar pattern of interaction is shown by *DPH5* (Figure 2B), *DPH2* and *DPH4* (data not shown). Based on correlated interaction profiles, FitDB ascribes GO terms enriched for processes concerning peptidyl-diphthamide biosynthesis from peptidyl-histidine to *YLR143w* and *YBR246w* with p-values of 2×10^−3^ and 9×10^−4^ respectively (Figure 2C). Collectively, the FitDB and DRYGIN profiles thus provide robust phenotypic signatures suggesting novel roles in the diphthamide pathway for *YBR246w* and *YLR143w*, which are tightly clustered within the *DPH* gene network (Figure 2C). This notion is consistent with a recent report that *YBR246w* and its mammalian homolog, WDR85, have a diphthamide related function [41], [42]. Since *YLR143w* is as yet unassigned in the *Saccharomyces* genome database (SGD), based on the evidence below that *YLR143w* and *YBR246w* are indeed diphthamide synthesis genes we have named them *DPH6* (*YLR143w*) and *DPH7* (*YBR246w*).

### *DPH6* and *DPH7* Deletions Cause Phenotypes Typical of *Bona Fide* Diphthamide Mutants

**Figure 3:**
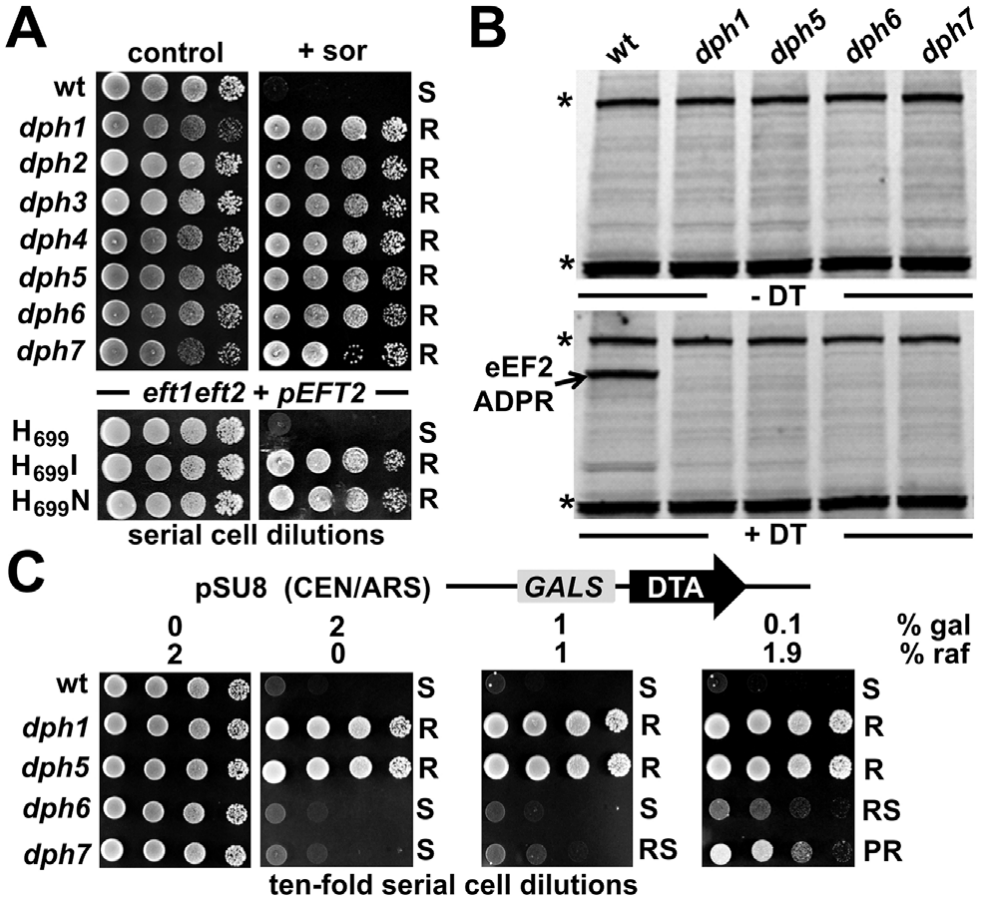
*DPH6* and *DPH7* deletion strains copy traits typically related to the *bona fide* diphthamide mutants *dph1-dph5*. (A) Sordarin resistance. Ten-fold serial cell dilutions of the indicated yeast strains, BY4741 wild-type (wt) background and its *dph1-dph7* gene deletion derivatives (upper panels) as well an MKK-derived *eft1 eft2* double deletion background maintaining plasmid p*EFT2* wild-type or H_699_ substitution (H_699_ N and H_699_I) alleles of *EFT2* (lower panels), were grown on YPD plates in the absence (control) or presence (+sor) of 10 µg ml^−1^ sordarin. Growth was assayed for 3 d at 30°C. Sordarin resistant (R) and sensitive (S) responses are indicated. (B) Lack of in vitro ADP ribose acceptor activity of eEF2. Cell extracts obtained from *dph1*, *dph5*, *dph6* and *dph7* mutant and wild-type (wt) strains were incubated with (+DT) or without (−DT) 20 nM diphtheria toxin in the presence of biotin-NAD (10 µM) at 37°C for 1 hour. The transfer of biotin-ADP-ribose to eEF2 was detected by Western blotting using a streptavidin-conjugate. Two unknown non-specific bands (indicated by *) served as internal controls for even sample loading. (C) DT phenotype. As indicated, yeast *dph* mutants and wild-type control (wt) were tested for sensitivity to intracellular expression of DTA, the cytotoxic ADP ribosylase fragment of DT. This in vivo assay involved galactose-inducible expression from vector pSU8 (see Materials and Methods). Serial cell dilutions were replica spotted onto raffinose (2% raf) and galactose-inducing conditions using concentrations (2, 0.2 and 0.1% gal) suited to achieve gradual down-regulation of DTA toxicity. Growth was for 3 days at 30°C. DTA sensitive (S) resistant (R), partially resistant (PR) and reduced sensitive (RS) phenotypes are indicated.

To verify the predicted roles for *DPH6* and *DPH7* in the diphthamide pathway, we next examined strains deleted for these ORFs for phenotypes specifically linked to defects in diphthamide formation on eEF2, namely sordarin resistance and response to DT [6], [7]. Sordarin traps eEF2 on the 80S ribosome [53], blocking mRNA translation elongation and yeast cell growth [54] in a fashion that depends on diphthamide synthesis [6], [7]. As a result, diphthamide mutants *dph1-dph5* efficiently protect against sordarin inhibition [6], [7]. Like *dph1-dph5*, *dph6* and *dph7* mutants showed robust resistance towards sordarin at 10 µg/ml, a concentration inhibitory to the wild-type (Figure 3A). This resistance was comparable to that shown by eEF2 substitution mutants *eft2*H_699_I and *eft2*H_699_N (Figure 3A), which lack the His_699_ acceptor residue for diphthamide modification [37]. Thus *DPH6* and *DPH7* are novel sordarin effectors, a feature they share with the diphthamide synthesis genes *DPH1-DPH5* [6], [7].

Diphthamide modification plays a key effector role for inhibitory ADP ribosylation of eEF2 by DT, hence *dph1-dph5* mutants in both yeast and mammalian cells confer resistance towards DT [14], [16]. We therefore compared DT-dependent ADP ribosylation of eEF2 in vitro between wild-type cells and *dph1*, *dph5*, *dph6* and *dph7* mutants. While the translation factor from wild-type cells was efficiently modified by the toxic ADP ribosylase (Figure 3B), eEF2 extracted from *dph1*, *dph5*, *dph6* and *dph7* mutants could not be ADP ribosylated by exogenously added DT under the conditions used (Figure 3B). Such lack of ADP ribose acceptor activity in vitro strongly suggests defects in the diphthamide pathway and that *DPH6* and *DPH7* encode novel functions required for diphthamide formation. To further address this experimentally, we assayed the response of *dph6* and *dph7* mutants to intracellular expression of the ADP ribosylase domain of DT (DTA) using *GALS*, a truncated variant of the *GAL1* promoter [55]. When DTA expression was induced by 0.1% galactose, *dph6* and *dph7* mutants were indeed found to show some protection against DTA in contrast to wild-type cells (Figure 3C), consistent with defects in diphthamide formation. However, at a higher level of expression on 2% galactose, they showed wild-type like sensitivity to DTA whereas *dph1* and *dph5* mutants remained fully resistant (Figure 3C). This suggests that eEF2 forms from *dph6* or *dph7* mutants, although not substrates in vitro (Figure 3B), can nonetheless be ADP ribosylated in vivo if DTA is expressed at a sufficiently high level [30]. While our work was in progress, eEF2 from a *ybr246w/dph7* mutant was shown to be a very weak substrate for ADP ribosylation when treated with 10 mM DT [42], a 500-fold increase in concentration over that used in our in vitro ADP ribosylation assays (Figure 3B). Thus eEF2 from the *dph6* or *dph7* mutants is resistant to sordarin and shows a vastly reduced ability to be ADP-ribosylated by DT, strongly suggesting that the diphthamide pathway is defective. Since the intermediate diphthine can serve as a sub-optimal substrate for ADP ribosylation using excess levels of DT or upon overexpressing its toxic ADP ribosylase domain from inside cells [29], [31], the properties of eEF2 from *dph6* and *dph7* mutants are consistent with a defect in the final step of the pathway that converts diphthine to diphthamide. Our analysis is therefore entirely consistent with the above database predictions and indicates *DPH6* and *DPH7* constitute novel candidate loci for diphthamide biosynthesis.

### Mass Spectrometry Reveals Diphthine Accumulation in *dph6* and *dph7* Mutants Due to a Block in the Terminal Amidation Step of the Diphthamide Pathway

Given the above evidence, we next examined whether eEF2 prepared from cells deleted for either *DPH6* or *DPH7* carried any modification on His_699_, the eEF2 residue that is modified to generate diphthamide. eEF2 preparations made from wild-type and gene deletion strains expressing His_6_-tagged eEF2 were digested with trypsin and examined by mass spectrometry. The His_6_-tagged form was chosen as the source of eEF2 since expression rescued the inviability of an *eft1 eft2* double mutant lacking eEF2 function, and it is thus considered to be biologically active [56]. Strains lacking either *DPH1*, in which the first step of diphthamide biosynthesis is blocked, or lacking *DPH5* (encoding diphthine synthase), were used respectively as controls for complete lack of modification and presence of ACP, the first intermediate in the diphthamide pathway [14], [16], [30], [32]. All strains expressed similar levels of His_6_-tagged eEF2 (data not shown).

**Figure 4:**
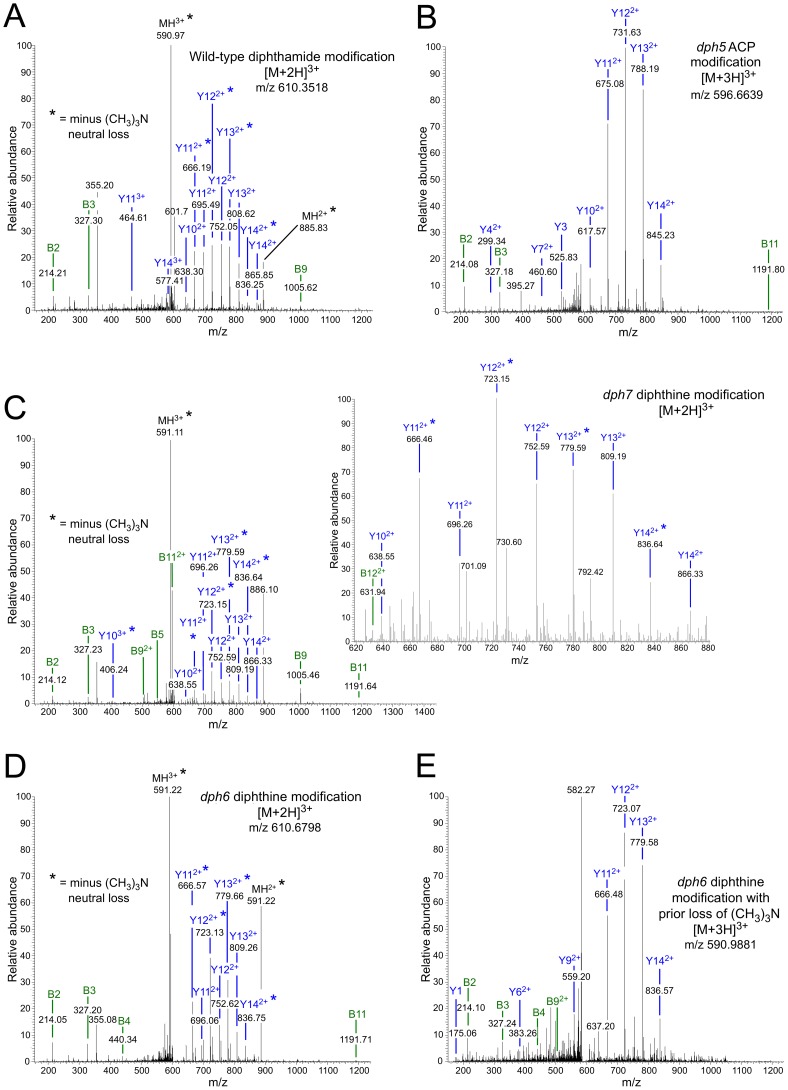
MS/MS spectra of diphthamide-, ACP-, and diphthine-modified EF2 peptide 686-VNILDVTLHADAIHR-700 from wild-type and mutant yeast strains. Spectra are shown for (A) diphthamide-modified peptide from the wild-type yeast strain; (B) ACP-modified peptide from the *dph5Δ* mutant; (C) diphthine-modified peptide in the *dph7Δ* strain; (D) diphthine-modified peptide in the *dph6Δ* strain; (E) diphthine-modified peptide in the *dph6Δ* strain with loss of the trimethylamino group before analysis in the mass spectrometer indicated by the parent ion m/z. In each case the parent ion m/z and charge state is indicated. In (A), (C) and (D), * indicates neutral loss of trimethylamino during MS/MS. The inset in (C) shows greater detail for the more crowded part of the MS/MS spectrum. Figure S2A indicates how the B and Y ions are derived from the peptide sequence.

The modified histidine in eEF2 (His_699_) is located in the tryptic peptide 686-VNILDVTLHADAIHR-700 and, as expected, unmodified versions of this peptide were readily detected in eEF2 prepared from the *dph1* mutant (Figure S1C). Unmodified peptide was also found in eEF2 prepared from *dph5*, *dph6* and *dph7* deletion strains as well as from wild-type cells (Figures S1 and S2). Thus even in wild-type cells not all of the eEF2 is modified by diphthamide. In addition to the unmodified peptide, we readily detected diphthamide-modified peptide in eEF2 prepared from the wild-type strain (Figure 4A), but failed to detect this in any of the mutants. Instead, ACP-modified peptide was found in eEF2 prepared from the *dph5* mutant (Figure 4B), as expected given its known role in generating diphthine [32] from the ACP intermediate in the pathway.

In contrast, eEF2 from the *dph7* mutant generated spectra consistent with the presence of diphthine on His_699_, in which the m/z values for both the parent ions and the daughter ions in the MS/MS spectra were higher in a manner consistent with the 0.984 Da extra mass associated with presence of a carboxyl group in diphthine rather than the amide group in diphthamide (Figure 4C). Thus each of the doubly-charged daughter ions in Figure 4C is larger by an m/z of ∼0.5 than the corresponding ion in the wild-type spectrum (Figure 4A). Furthermore, the quite different elution times of the diphthine-modified and diphthamide-modified peptide that are evident from the extracted ion chromatograms (Figure S3) are consistent with differently modified forms of eEF2. As noted in previous studies [32], [33], [36], some of the ions in our MS/MS spectra had undergone neutral loss of the trimethylamino group during MS/MS, as indicated by loss of 59.110 mass units.

Two types of spectra corresponding to the peptide with modified His_699_ were seen when eEF2 from the *dph6* mutant was analyzed. In some spectra (Figure 4D), the parent ion m/z and MS/MS data indicated the presence of diphthine as in the *dph7* mutant, with some daughter ions again showing neutral loss of the trimethylamino group during MS/MS as noted above. However, we also detected peptide forms in which elimination of the trimethylamino group had occurred prior to analysis, as indicated by the lower parent ion m/z (Figure 4E) and an MS/MS spectrum in which all assignable peaks corresponded to ions lacking the trimethylamino group. Such trimethylamino elimination prior to mass spectrometry was observed previously when diphthine-modified *Pyrococcus horikoshii* EF2 was generated in an in vitro reaction [32], indicating that this modification might be unstable. However, we failed to detect any pre-mass spectrometry loss of the trimethylamino group when eEF2 from the *dph7* mutant was analyzed. Thus while eEF2 from both mutants carries diphthine, the modification appears to be more labile in the *dph6* mutant and may be protected from trimethylamino elimination by the absence of Dph7.

Figure S3 shows extracted ion chromatograms for ions with m/z values corresponding to the His_699_ containing peptide modified with diphthamide, diphthine or with ACP, indicating that the ACP modified peptide was only present in the *dph5* mutant, the diphthine modified peptide was only present in *dph6* and *dph7* mutants, and diphthamide-modified peptide was only seen in wild-type cells. Our mass spectrometry analysis therefore shows that in yeast strains lacking either *DPH6* or *DPH7*, modification of His_699_ progresses only as far as diphthine. Thus both loci indeed qualify as novel diphthamide synthesis genes with likely roles in conversion of diphthine to diphthamide.

### Protein–Protein Interactions Between Dph6, Dph7, Dph5, and EF2

**Figure 5:**
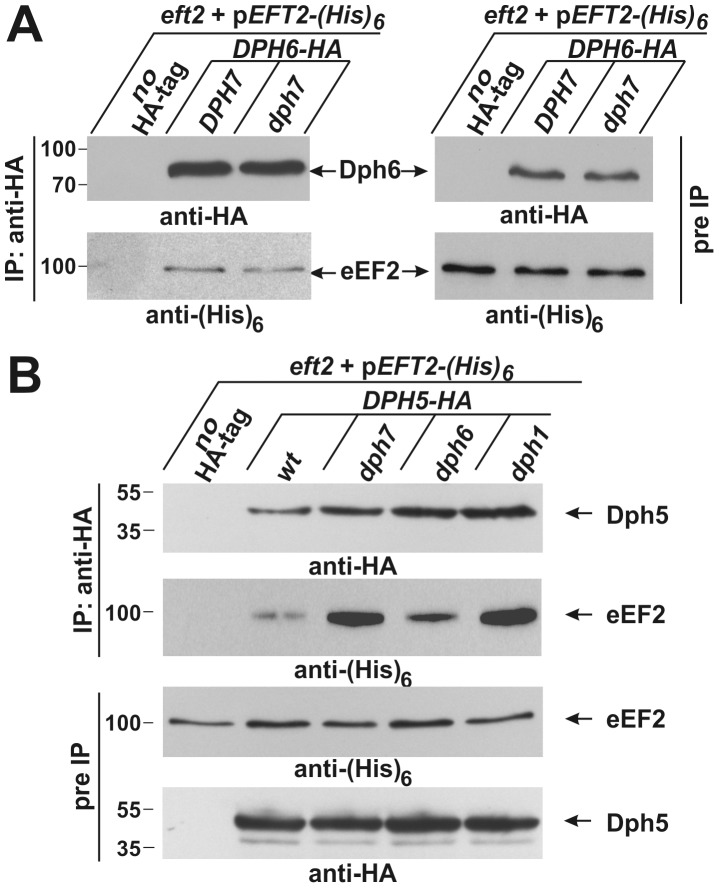
Co-immune precipitations reveal eEF2 interactions with Dph6 and Dph5. (A) eEF2 interacts with Dph6 in a fashion that is independent of Dph7. (B) eEF2 interaction with Dph5 is dramatically enhanced by elimination of Dph7 or Dph1. Yeast strains co-expressing (His)_6_-tagged eEF2 with Dph6-HA (A) or Dph5-HA (B) in the background of wild-type (A: *DPH7* and B: wt) and *dph* mutant strains (A: *dph7*; B: *dph1*, *dph6* and *dph7*) were subjected to immune precipitations (IP) using the anti-HA antibody. Strains expressing (His)_6_-tagged eEF2 on their own served as IP controls (A and B: no HA-tag). Subsequently, the precipitates were probed with anti-HA (A: top left panel; B: first panel) and anti-(His)_6_ antibodies (A: bottom left panel) to check for the content of Dph6-HA (A) and Dph5-HA (B), respectively (all indicated by arrows). The content of HA-tagged Dph6 (A) and Dph5 (B) as well as (His)_6_-marked eEF2 (A and B) in the protein extracts prior to IP (pre-IP) was examined on individual Western blots using anti-HA (A: top right panel; B: fourth panel) and anti-(His)_6_ antibodies (A: bottom right panel; B: third panel), respectively. While absence of Dph7 hardly affected the Dph6•eEF2 interaction (A), Dph5•eEF2 interaction was strongly enhanced by inactivating *DPH7* or *DPH1* (B).

Although Dph6 and Dph7 appear to function within the same step of the diphthamide synthesis pathway, using co-immune precipitation they were not found to interact either with one another or with Dph2 and Dph5, players involved in the two earlier pathway steps (Figure S4; Figure S5 and data not shown). However, in support of our evidence that Dph6 is a diphthamide biosynthetic factor, we observed by co-immune precipitation that Dph6-HA bound to a fraction of (His)_6_-tagged eEF2 (Figure 5A). Intriguingly, this interaction was independent of Dph7 (Figure 5A), suggesting Dph7 may not mediate interaction between Dph6 and the translation factor. Dph7 is also unlikely to play an indirect role through regulation of *DPH6* gene expression because Dph6 protein levels were unaltered in the *DPH7* deletion strain (Figure 5A).

**Figure 6:**
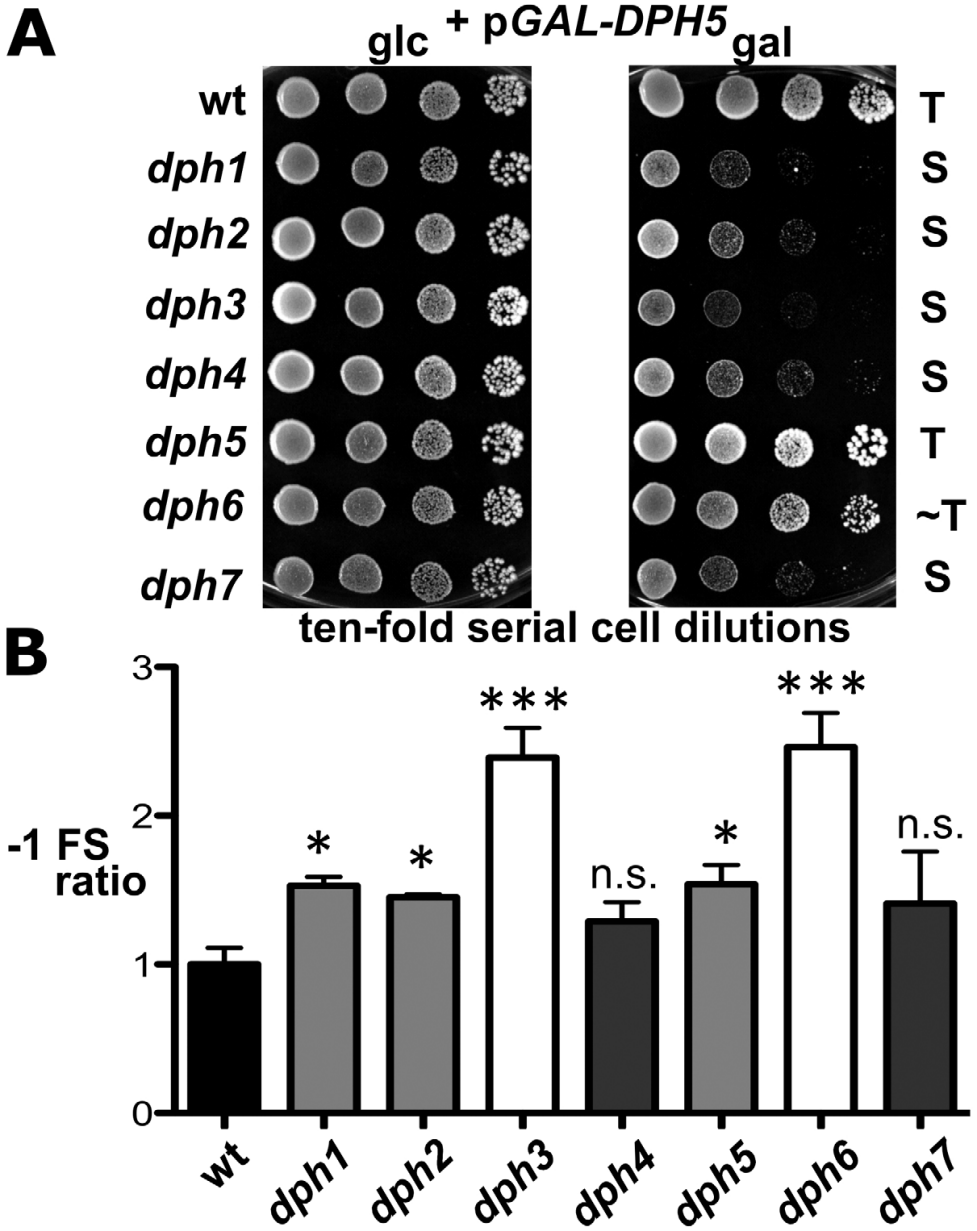
*dph* mutants show sensitivity to elevated diphthine synthase levels and confer reduced translational accuracy. (A) *DPH5* overexpression in *dph1-dph4* and *dph7* mutants causes cytotoxicity and a severe cell growth defect. Cells of yeast strains with the indicated genetic backgrounds and maintaining plasmid p*GAL-DPH5* for galactose inducible overexpression of diphthine synthase Dph5 were serially diluted and replica spotted onto glucose (2% glc) and galactose (2% gal) media to assay their response to *DPH5* overexpression. Growth was for 3 days at 30°C. Unaltered (T), slightly weakened tolerance (∼T) and sensitive (S) responses are indicated. Note that *dph1-dph4* and *dph7* mutants are extremely sensitive to *DPH5* overexpression. (B) Ribosomal frameshift analysis reveals erroneous translation in *dph1-dph7* mutants. Strains with the indicated genetic backgrounds were transformed with control (pJD240.0) or *lacZ* −1 frameshift (pJD240.−1) plasmids [59] to monitor *lacZ* expression through β-galactosidase (β-Gal) production using *O*-nitrophenol-D- galactopyranoside assays and to score translation efficiency (pJD240.0) and fidelity (pJD240.−1). Ribosomal −1 frameshifts are expressed relative to the level of overall translation efficiency with statistical significance determined by one-way ANOVA followed by Dunnett's multiple comparison. With the exception of *dph4* and *dph7*, post-hoc comparison found that all other mutant backgrounds showed a significant increase in ribosomal −1 frameshifting relative to wild-type (wt) yeast cells (* = P<0.05; *** = P<0.001; ns. = not significant).

Inactivation of WDR85, the mammalian homolog of Dph7, was recently shown to dramatically enhance association of diphthine synthase Dph5 with eEF2 [41]. We therefore examined whether Dph7 impacts on the interaction between Dph5 and eEF2 in budding yeast. We found that a much higher level of affinity tagged eEF2 could be co-immune precipitated with HA-tagged Dph5 from extracts of the *dph7* mutant in comparison to wild-type extracts (Figure 5B). A smaller increase was also seen with the *dph6* mutant (Figure 5B). This strongly suggests a conserved role for Dph7/WDR85 as a regulator of the Dph5•eEF2 interaction. Remarkably, we also found similarly enhanced binding of Dph5 to eEF2 in the *dph1* mutant, which has a defect in the first step of the diphthamide pathway and therefore lacks the ACP modification that is the immediate substrate of diphthine synthase (Figure 5B). Strikingly, *DPH5* overproduction from a galactose-inducible promoter was found to be highly detrimental to cells deleted for *DPH7* and to all mutants blocked at the first step of the pathway, but had little effect on the *dph6* mutant and no effect on wild-type or *dph5* cells (Figure 6A). Intriguingly, this cytotoxicity goes hand in hand with the enhanced Dph5•eEF2 interaction profiles we observed in *dph1*, *dph6* and *dph7* cells under conditions of wild-type *DPH5* copy number and normal Dph5 expression levels (Figure 5B). Taken together, our results suggest that binding of Dph5 to incompletely modified eEF2 may be inhibitory to the function of the translation factor. Our data also indicate that both unmodified eEF2, and diphthine-modified eEF2 in the absence of Dph7, show strongly enhanced binding to Dph5. Furthermore, since we failed to detect association between Dph5 and Dph6 despite demonstrating interaction of each with eEF2, it is likely that Dph5 and Dph6 do not bind concurrently to eEF2 and that their binding may therefore be mutually exclusive.

### Physiological Implications of the Diphthamide Modification on eEF2

Although the precise biological function of diphthamide is unclear, its location at the tip of the eEF2 anticodon mimicry domain IV predicts a potentially important role in translation. Consistent with this, structure-function studies have shown that domain IV is sufficiently proximal for interaction with tRNA in the decoding P-site of the ribosome [57] and alterations of invariant tip residues, including H_699_ substitutions that cannot be diphthamide modified, confer biologically significant traits including thermosensitive growth defects [37], [58]. Nonetheless, when compared to their wild-type parental strain, we found no significant changes in the growth performance of *dph1-dph7* mutants in either liquid or on solid media and at standard cultivation temperatures of 30°C (Figure S6). Even increasing the cultivation temperatures to 39°C had no discernable effect on *dph* cell growth except for the *dph3/kti11* mutant (Figure S6), which is known to be thermosensitive due to additional functions unrelated to diphthamide [6].

However, intrigued by previous reports that diphthamide defects can induce ribosomal frame-shifts [6], [36], we next studied whether *DPH6* and *DPH7* deletions affect the accuracy of eEF2 in the translation process (Figure 6B). Using *lacZ*-based reporters to monitor programmed +1 and −1 frameshift signals derived from Ty elements [36], [59], *dph1-dph7* mutants failed to induce significant ribosomal +1 frameshifts (data not shown). However, *dph1*, *dph2*, *dph3*, *dph5* and *dph6* mutants significantly enhanced *lacZ* expression dependent on a −1 frameshift, with *dph6* and *dph3* cells scoring as the top −1 frameshifters followed by lower but statistically significant effects in *dph1*, *dph2* and *dph5* mutants (Figure 6B). This confirms increased −1 frameshifting in *dph2* and *dph5* mutants seen previously [36] and demonstrates an even larger defect in *dph3* and *dph6* strains. Ribosomal −1 frameshift induction by *dph7* and *dph4*, though slightly increased in relation to wild-type controls, was considered statistically insignificant (Figure 6B). The −1 frameshifting phenotype shared between *dph6* and *bona fide dph* mutants is consistent with a role for diphthamide in promoting translational accuracy of eEF2. In line with a role for diphthamide in the fine tuning of translation elongation, growth assays performed under thermal and/or chemical stress conditions showed that certain *dph* mutants including *DPH6* and *DPH7* deletion strains displayed altered responses to translation elongation indicator drugs such as hygromycin, anisomycin or paromomycin (Figure S7). In conclusion, our data indicate that diphthamide mutant strains such as *dph6* increase ribosomal errors typical of −1 translational frameshifts and that the diphthamide modification function of Dph6, which is required for completion of diphthamide synthesis, is likely to assist eEF2 in reading frame maintenance during translation.

## Discussion

We have presented genetic, phenotypic, mass spectrometric and biochemical analyses that clearly identify Dph6 as a novel protein required for the final step of diphthamide biosynthesis and that confirm a similar role for Dph7 as reported recently [41], [42]. Thus in yeast strains lacking either *DPH6* or *DPH7*, modification of His_699_ on eEF2 progresses only as far as diphthine and these gene products are required for amidation of diphthine to generate diphthamide. Our findings are consistent both with a recent bioinformatics analysis that predicted a role for Dph6 in the diphthine to diphthamide conversion [60] and with the identification of Dph6 as yeast diphthamide synthetase reported by Su et al. [61] while we were revising our manuscript.

### Dph6

**Figure 7:**
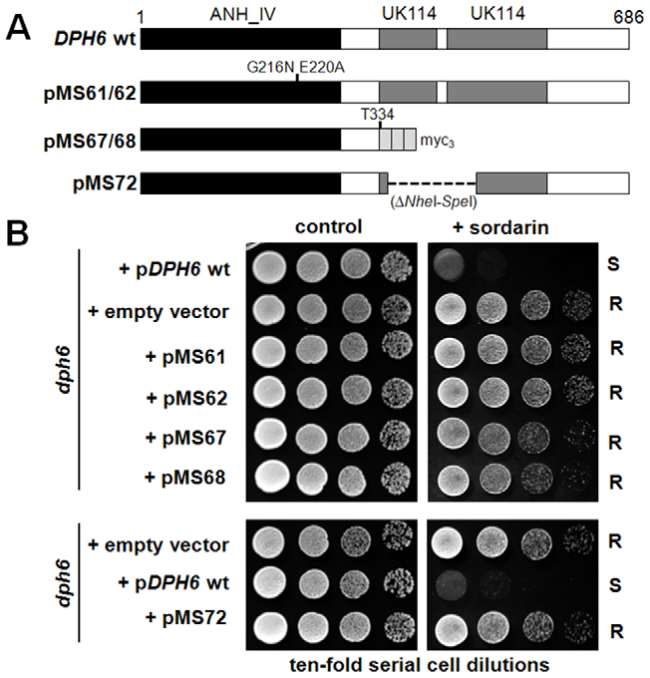
Both the Alpha_ANH_like_IV and YjgF-YER057c-UK114 domains in Dph6 are essential for its functionality. (A) Diagram showing the *DPH6* wild-type and mutant constructs tested in (B), indicating the Alpha_ANH_like_IV (ANH) and YjgF-YER057c-UK114 (UK114) domains and the position of point mutations, an in-frame deletion (- - - - -) and triple myc epitope tag (*myc*_3_) as appropriate. (B) Ten-fold serial cell dilutions of a *dph6* deletion strain carrying the constructs shown in (A) or the corresponding empty vector (top panel, pSU6 [wt *DPH6*]; lower panel, pSU7 [wt *DPH6*]: Table S3) were spotted onto SCD-Leu plates with or without 10 µg/ml sordarin and grown at 30°C for 3 days.

Dph6 contains three conserved domains consistent with it functioning as an enzyme (Figure S8). The amino-terminal 225 residues constitute an Alpha_ANH_like_IV domain (cd1994 in the NCBI Conserved Domain Database [62], also known as DUF71), a member of the adenine nucleotide alpha hydrolase superfamily that is predicted to bind ATP. Many DUF71 proteins from archaea to mammals contain the highly conserved motif –E_215_GG(D/E)XE_220_– (Dph6 numbering), which has been proposed to be involved in substrate binding and catalysis and which is replaced by –ENGE(F/Y)H– in a group of related DUF71 proteins implicated in biotin synthesis [60]. Based on this we generated a *dph6* allele encoding two substitutions in this region (G216N, E220A) and tested its functionality by monitoring complementation of sordarin resistance in a yeast *dph6* knockout strain. Figure 7 clearly shows that this small change completely inactivates the function of Dph6, demonstrating that the Alpha_ANH_like_IV domain is critical for the conversion of diphthine to diphthamide. The C-terminal portion of Dph6 contains two domains related to the YjgF-YER057c-UK114 protein family (eu_AANH_C1: cd06155 and eu_AANH_C2: cd06166) that may promote homotrimerisation and formation of an inter-subunit cleft that has been proposed to bind small molecule ligands [63]–[65]. Several key residues in human UK114 required for homotrimerisation and ligand binding [66] are present in Dph6 (Figure S8) including arg-107, which in *E. coli* TdcF forms a bidentate salt bridge with the carboxylic acid group of bound ligands [63]. Deletion of residues 335–415 encompassing much of the YjgF-YER057c-UK114 region abolished the function of Dph6 as monitored by sordarin resistance (Figure 7), while truncation of Dph6 at the first of the two conserved domains by insertion of a *myc* tag also eliminated Dph6 function (Figure 7) despite detectable expression of the truncated polypeptide (data not shown), indicating that the YjgF-YER057c-UK114 domains are also important for Dph6 function and that the Alpha_ANH_like_IV domain is nonfunctional on its own. Since *Salmonella enterica* YjgF has an enamine/imine deaminase activity that is conserved in human UK114 [67] it is possible that the YjgF-YER057c-UK114 domains in Dph6 are used to generate ammonia for diphthamide formation.

Taken together, these properties suggest a direct, ATP-dependent role for Dph6 in diphthine amidation proceeding via an adenylated intermediate and with ammonia acting as the source of the amide group. Such a direct role has now been demonstrated by Su et al., who have used an in vitro assay system to show that Dph6 has diphthamide synthetase activity [61]. Although proteins showing Dph6-like domain organization are readily identified in fungi, plants, amphibians and insects (Figure S8), they are largely absent from archaeal and mammalian proteomes. However, mammals and archaea have separate proteins showing strong similarity to either the adenine nucleotide alpha hydrolase domain or to the YjgF-YER057c-UK114 related regions (Figure S8 and data not shown), suggesting Dph6 functionality may be split between different polypeptides in these cases. It is therefore surprising that expression of the human *DPH6* ortholog in a yeast *dph6* mutant can restore diphthamide biosynthesis [61] despite lacking the YjgF-YER057c-UK114 domains that are essential in the yeast protein (Figure 7; [61]). Thus while the core function of the enzyme must therefore reside in the Alpha_ANH_like_IV domain, it will be interesting to determine the role of the YjgF-YER057c-UK114 domains in Dph6 from lower eukaryotes.

### Dph7

**Figure 8:**
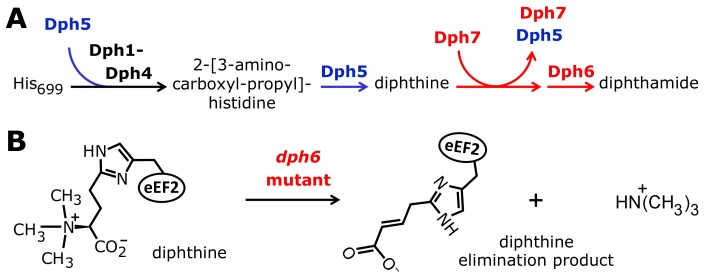
Model for the diphthamide pathway incorporating the proposed novel roles of Dph5, Dph6, and Dph7. (A) Diphthamide pathway showing interaction of Dph5 with unmodified eEF2 and the proposed role of Dph7 in displacement of Dph5 prior to diphthine amidation. (B) Elimination of the trimethylamino group in the absence of the proposed amidase Dph6 suggesting lability of diphthine in its absence.

Dph7 has four well-defined WD40 repeats (Figure S9) and its predicted structure consists exclusively of β-sheet elements [41], [68]. Although its human homolog WDR85 has been implicated in the first step of diphthamide biosynthesis [41], our work and that of Su et al. [42] show that the pathway can proceed as far as diphthine in the absence of *DPH7* and that the block is therefore in conversion of diphthine to diphthamide. Furthermore, this block cannot be bypassed simply by introducing *DPH6* on a multicopy plasmid to increase the level of diphthamide synthetase (data not shown). How then might Dph7 contribute to diphthine amidation? Its domain structure suggests it could act as an adaptor molecule for diphthine amidation [42], but this notion is at odds with our failure to detect interaction between Dph7 and Dph6 (see above). Our intriguing finding that eEF2 binds much more Dph5 in the absence of Dph7 suggests an alternative role, namely that Dph7 is needed to displace Dph5 from diphthine-modified eEF2 to allow the amidation reaction to occur. Similar findings in mammalian cells upon inactivation of WDR85 support this notion [41]. Together with our data showing that viability of *dph1-dph4* and *dph7* cells is extremely sensitive to excess Dph5 in comparison to wild-type or *dph6* cells, it appears that binding of Dph5 to eEF2 is inhibitory to the function of the translation factor and negatively interferes with cell growth unless eEF2 carries the completed diphthamide modification. Perhaps in addition to catalyzing methylation of ACP-modified eEF2, Dph5 binds to newly-synthesised eEF2 to exclude it from functioning in translation until the diphthine amidation step takes place (Figure 8). Consistent with this proposal is our observation that the level of Dph5 associated with eEF2 in the *dph1* mutant, in which modification of His_699_ cannot be initiated, is drastically increased and virtually indistinguishable from the enhanced Dph5-eEF2 interaction seen when Dph7 is absent. Dph7 may be needed to displace Dph5 once diphthine has been generated so that Dph6 can carry out the diphthine to diphthamide conversion (Figure 8), a notion consistent with the sensitivity of the *dph7* mutant to *DPH5* overexpression. In contrast, the *dph6* mutant may tolerate Dph5 overexpression because Dph7 is present to displace it.

Two other seemingly unrelated functions have been previously proposed for *DPH7*. Firstly, it emerged from a genetic screen as a potential negative regulator of RNA polymerase I (Rrt2), although no other *DPH* genes were similarly identified [69]. Secondly, *DPH7* has been implicated in retromer mediated endosomal recycling and named *ERE1* [68]. The connection between endosomal recycling and diphthamide biosynthesis is currently unclear and it remains to be determined whether Dph7 is multifunctional or if these other roles are linked to its eEF2 modification function.

Diphthamide on eEF2 is the target for bacterial ADP-ribosylase toxins and also affects toxicity of sordarin and ricin, a ribosome inhibiting protein toxin from plants [70]. Although this emphasizes its pathological relevance, the physiological significance of diphthamide remains enigmatic and elusive. Nonetheless, the evolutionary conservation of the diphthamide pathway among eukaryotes and the embryonic lethality of mice that cannot synthesize diphthamide [38] strongly suggest that it is important in translation related processes. In support of this notion, evidence presented here and by others shows that diphthamide mutants cause increased translational frameshifting, a defect also observed in mammalian cells [6], [36], [71]. Diphthamide modification may have particular importance in multicellular organisms or when cells are stressed [4]. Mutation of mammalian diphthamide synthesis genes affects cell proliferation and development: inactivation of *DPH3/KTI11* is associated with tRNA modification defects and neurodegeneration and mutations in *DPH1/OVCA1* revealed a tumor suppressor role for this diphthamide synthesis gene in ovarian cancer [27], [38]–[40], [72]. Regardless of its physiological functions, our data indicate that the diphthamide pathway is more complex than originally anticipated and comprises, in addition to Dph1-Dph5, two further components, Dph6 and Dph7, which operate in the terminal amidation step (Figure 8). While it is now clear that Dph6 is diphthamide synthetase [61], in the future it will be important to understand why the archaeal and mammalian orthologs can dispense with the otherwise conserved YjgF-YER057c-UK114 domains and to define the precise role of Dph7. It will also be crucial to explore the potential role of diphthine synthase (Dph5) as a potential regulator of the entire pathway and the reasons for apparent lability of diphthine in the *dph6* mutant that is suggested by our data (Figure 8).

## Materials and Methods

### Strains, Media, Growth Conditions, and Growth Assays

Yeast strains used in this study are listed in Table S2 and plasmids in Table S3. Cultures were grown in complete (YPD) or minimal (SD) media [73] at 30°C unless otherwise stated. For phenotypic assays, YPD was supplemented with 10 µg/ml sordarin sodium salt from *Sordaria araneosa* (Sigma-Aldrich). Yeast transformations with plasmid DNAs were performed following the lithium acetate protocol [74]. Diphtheria toxin (DT) growth assays in vivo involved expression of the toxin's cytotoxic ADP ribosylase fragment (DTA) from vector pSU8 (p415-*GALS*-DTA), essentially as previously described for *dph1-dph5* mutants [6]. pSU8 was made by cloning the *Bam*HI fragment encoding DTA from pLMY101 [30] into plasmid p415*-GALS*, a single-copy *E. coli*-yeast shuttle vector with a truncated *GAL* promoter [55], which allows for conditional DTA induction on galactose-containing media. [55]. The translational frameshift reporter assay essentially involved previously published protocols together with the described *lacZ* reporter plasmids pJD204.0 (wild-type control), pJD204.−1 (−1 frame) and pJD204.+1 (+1 frame) [36], [59]; the pJD204 plasmid series was kindly provided by T. Kinzy (UMDNJ, USA). The relative values for +1 and −1 frameshifting were statistically analyzed using one-way ANOVA followed by Dunnett's multiple comparison post test and was performed with Graphpad Prism 5.0 software essentially as previously described [75].

### Gene Deletion and Epitope Tagging

Details of all primers used in numerous PCR dependent genomic manipulation experiments can be found in Table S4. Gene deletions were performed using in vivo PCR-based one step-gene disruption protocols in combination with marker plasmids YDpKl-L, YDpKl-U or YDpSp-H [76] and knockout primers (Table S4) including those previously described [6], [25], [77]. Gene deletions were confirmed via diagnostic PCR on genomic DNA preparations using target ORF-specific primer pairs (Table S4) as well as sordarin response assays. C-terminal tagging of *DPH1*, *DPH2*, *DPH5*, *DPH6/YLR143w* and *DPH7/YBR246* was performed according to previously published in vivo PCR-based epitope tagging protocols [78] using appropriate S3/S2 primer pairs (Table S4). Tagged genes were confirmed by Western blot detection with anti-HA or anti-c-Myc antibodies (Santa Cruz Biotechnology A-14 and F7, respectively). Detection of HA- or c-Myc-tagged Dph1, Dph2, Dph5, Dph6 and Dph7 as well as Dph3 and Elp2 in co-immune precipitation (Co-IP) assays were performed as previously described [6], [77], [79].

### *DPH6* Constructs

pSU6 was generated by insertion into YCplac111 [80] of a genomic PCR fragment including *DPH6* together with 829 bp of upstream and 59 bp of downstream sequence flanked by *Eco*RI and *Bam*HI sites incorporated using PCR primers (Table S4). The insert was verified by sequencing and shown to complement a *dph6* knockout strain. pSU7 was made by cloning the *DPH6* insert from pSU6 into YEplac181 [80]. To generate a G216N E220A *dph6* mutant, pSU6 was digested with *Age*I and *Bsm*BI and the small *DPH6* fragment replaced by an identical synthetic fragment (Integrated DNA Technologies) carrying the G216N E220A mutations, generating independent clones pMS61 and pMS62. The replaced region was verified by DNA sequencing. pMS67 and pMS68 were generated from pSU6 by replacing the *Bsm*BI-*Sal*I fragment carrying the C-terminal region of *DPH6* and downstream sequence with a synthetic *Bsm*BI-*Sal*I fragment in which codons 335–685 were replaced by sequence encoding the linker and triple *myc* tag from pYM23 [81]. To generate pMS72, the smaller *Nhe*I-*Spe*I fragment of pSU7 was excised and the large fragment ligated to generate an in-frame fusion that removed *DPH6* codons 347–471, checking the resulting fusion by DNA sequencing.

### In Vitro ADP Ribosylation Assay

Yeast cell extracts were prepared as described previously [15]. ADP ribosylation reactions were performed at 37°C for 1 hour in a volume of 40 µl ADP ribosylation buffer (20 mM Tris-HCl, pH 7.4, 1 mM EDTA, 50 mM DTT) containing 50 µg of yeast extract, 50 ng fully-nicked DT and 10 µM 6-biotin-17-NAD (Trevigen). Samples were then mixed with SDS sample buffer, boiled for 5 min and run on 4–25% SDS-PAGE gradient gels (Invitrogen). The proteins were transferred to nitrocellulose membranes and Western blotting was performed using streptavidin-IR conjugate (Rockland Immunochemicals, Gilbertsville, PA) and scanned on an Odyssey Infrared Imager (LICOR Biosciences, Lincoln, NE).

### Expression and Purification of Affinity-Tagged eEF2-(His)_6_

BY4741 wild-type yeast cells as well as *dph1*, *dph5*, *ylr142w/dph6* and *ybr246w/dph7* mutants thereof carrying an *eft2* null-allele were transformed with plasmid pTKB612 (a kind gift from A. R. Merrill, University of Guelph, Ontario, Canada), which expresses a (His)_6_-tagged form of translation elongation factor eEF2 (Table S3) that is fully functional and able to complement an *eft1 eft2* double mutant [56]. In order to express and purify (His)_6_-tagged eEF2 for MS/MS analysis, 750 ml of yeast culture were grown in YPD to an OD_600_ 2.0 and harvested by centrifugation. The pellet was resuspended in 3 ml B60 buffer (50 mM HEPES-KOH pH 7.3, 60 mM KOAc, 5 mM Mg(OAc)_2_, 0.1% Triton X100, 10% (v/v) glycerol, 1 mM NaF, 20 mM glycerophosphate, complete protease inhibitor [Roche]) without DTT and cells were lysed in a bead beater. The lysate was centrifuged twice at 13,500 rpm for 30 min. and the protein concentration measured with a NanoDrop spectrophotometer. Five mg total protein was applied to 2 mg anti-(His)_6_-tag Dynabeads (Invitrogen, #101-03D) and purified according to manufacturer's instructions. The identity of purified eEF2 fraction was confirmed by SDS-PAGE and Western blot analysis using an anti-(His)_6_ antibody (Abcam, #ab18184).

### Analysis of Diphthamide Pathway Modifications on eEF2 by Mass Spectrometry

Crude yeast eEF2 preparations from wild-type and *dph* mutants strains were separated by SDS-PAGE using 4–12% Bis-Tris precast gels (Invitrogen, Carlsbad, USA) and the area of the gel containing eEF2 was excised after staining with Instant Blue Coomassie (Expedeon, Cambridge, UK). In-gel digests were performed using trypsin, subsequent to reduction and alkylation with dithiothreitol and iodoacetamide, with the resulting peptides cleaned over C18 columns. Peptides were then analyzed via HPLC-MS/MS using a Dionex U300 HPLC (Dionex California) with a 15 cm PepMap C18 column coupled to a Thermo Orbitrap Velos mass spectrometer (Thermo Fisher Scientific, Bremen, Germany). The peptides were eluted from the C18 column at 300 nL/min over 120 min using a linear 5–90% (v/v) acetonitrile gradient. The Orbitrap Velos was operated in positive ion mode, with an ion source voltage of 1.2 kV and capillary temperature 200°C, using a lock mass of 445.120024. The initial survey scan was performed at 60000 resolution, FTMS scanning from 335–1800 Da. The top 15 most intense ions were selected for MS/MS sequencing, using collision-induced dissociation (CID; MS/MS charge state 1+ rejected, >2+ accepted). Protein identification was performed using MaxQuant 1.2.2.5 [82] against a proteome database generated from the *Saccharomyces* Genome database [83]. Manual annotation of the modified peptide spectra corresponding to the modified EF2 peptide and generation of extracted ion chromatograms were done using the Thermo Xcalibur software for spectra visualization.

## Supporting Information

Figure S1 MS/MS spectra of unmodified eEF2 peptide 686-VNILDVTLHADAIHR-700 from wild-type and mutant yeast strains. (A) Cartoon showing how the B and Y ions seen in the MS/MS spectra map onto the tryptic peptide containing His-699. Y1 to Y13 and B14 ions contain His-699 and their m/z values are therefore informative regarding the modification state of His-699. (B–F) MS/MS spectra of unmodified peptide in eEF2 obtained from the indicated yeast strains: the parent ion m/z and charge state is indicated in each case.
(TIF)

Figure S2 Extracted ion chromatograms of unmodified EF2 peptide 686-VNILDVTLHADAIHR-700. In (A), peaks corresponding to doubly-charged ions (m/z unmodified peptide 843.97, extracted mass range 843.8–844.0) are shown while triply-charged ions (m/z unmodified peptide 562.98, extracted mass range 562.5–563.2) are shown in (B). The yeast strain to which each chromatogram pertains is indicated. Note that in (B) an intensity of 580,000 corresponding to unmodified peptide with m/z 562.98 was not resolved from a different, more abundant ion with m/z 563.02 in the wt sample. Peak annotations are as follows: RT, retention time; AA, peak area; BP, parent ion m/z.
(TIF)

Figure S3 Extracted ion chromatograms of modified eEF2 peptide 686-VNILDVTLHADAIHR-700. (A) Peaks corresponding to triply-charged ions (m/z diphthine-modified peptide 610.68, m/z diphthamide-modified peptide 610.35, extracted masses 610.2–610.9). (B) Triply-charged ions (m/z ACP-modified peptide 596.66, extracted masses 596.2–596.8). Peak annotations are as follows: RT, retention time; AA, peak area; BP, parent ion m/z.
(TIF)

Figure S4 Failure to detect interaction by TAP-based co-immune precipitation between Dph6 or Dph7 and either Dph2 or diphthine synthase Dph5, factors integral to the first two steps of diphthamide synthesis. Co-immune precipitations were performed using magnetic beads (Dynabeads, Invitrogen) coupled to anti-CBP antibodies (Santa Cruz Biotechnology) specific for the calmodulin binding peptide (CBP) of the TAP-tag. The indicated strains expressed *DPH6-TAP* or *DPH7-TAP* in conjunction with HA-tagged versions of either *DPH2* or *DPH5*. A strain co-expressing respectively, HA- and TAP-tagged variants of Dph1 and Dph3, step 1 pathway players previously shown to associate with one another [6], [20] served as a positive internal control for interaction. The presence of the respective proteins within the immune precipitates (IP) was assessed using anti-HA and anti-CBP Western blots (A) or anti-HA immune blots on total protein extracts obtained prior to the IP protocol (preIP). (B). Asterisks indicate breakdown products of Dph2-HA, Dph3-TAPand Dph6-TAP.
(TIF)

Figure S5 Failure to detect Dph6-Dph7 interaction by co-immune precipitation. Co-immune precipitations using the anti-HA-antibody were performed with the indicated strains expressing *DPH6-c-myc* or *DPH7-c-myc* on their own or in parallel with HA-tagged versions of *DPH5* or *DPH6*, respectively. A strain co-producing c-Myc- and HA- and tagged versions of the Elp2 subunit (*ELP2-c-myc*) of the Elongator complex, and Kti12 (*KTI12-HA*), a protein known to interact with Elp2 [84], was used as internal positive control. The presence of the respective proteins was assessed in individual anti-c-Myc and anti-HA Western blots both in the IPs (top two panels) and crude extracts (pre IP; bottom two panels). The asterisk denotes an unspecific band that originates from the anti-HA-antibody present in the IPs.
(TIF)

Figure S6 Lack of effect of *dph1-dph7* gene knockouts on growth performance and viability. (A) The wild-type parental strain and diphthamide deficient mutants *dph1*, *dph6* and *dph7* were grown in YNB minimal media supplemented with His, Met, Ura, Leu to cover the auxotrophic markers (Table S2) under standard laboratory conditions over a period of 50 h. OD_600_ was monitored at 2 h intervals. (B) To address a potential temperature sensitive phenotype, ten-fold serial cell dilutions of the indicated strains were spotted on YPD plates and grown at 30°C or 39°C. Note that only the *dph3/kti11* mutant, which affects additional biosynthetic pathways [6], [85] apart from diphthamide biosynthesis [13] shows temperature sensitivity (S) (S) while the other *dph* mutants tolerate high temperatures (T).
(TIF)

Figure S7 Altered growth performance of *dph1-dph7* mutants in response to translation elongation indicator drugs under standard or elevated cultivation temperatures. Ten-fold serial cell dilutions of wild-type parental strain as well as diphthamide mutants *dph1-dph7* were replica spotted on YPD plates without (control) and supplemented with hygromycin (20 µg/ml), anisomycin (20 µg/ml) or paromomycin (1.5 mg/ml) and grown at 30°C (A) or 37°C (B). Reduced or improved performance of the *dph* mutants relative to wild-type behavior reflects respectively, enhanced sensitivity or improved tolerance towards the drug in question respectively.
(TIF)

Figure S8 Conservation of the *DPH6* gene product, Dph6. (A) Representation of Dph6 indicating the conserved adenine nucleotide alpha hydrolase (cd1994) and YjgF-YER057c-UK114 related (cd06155, cd06166) domains discussed in the main text. (B) The Dph6 amino acid sequence was aligned using Clustal with representative examples of putative orthologs from other organisms (identified by PSI-BLAST). Sequences are as follows (database accession numbers in parentheses): *DPH6*, *S. cerevisiae* Dph6/Ylr143w; Sp_mug71, *Schizosaccharomyces pombe* (NP 595310); At_A_AAH_IV, *Arabidopsis thaliana* endoribonuclease (NP 187098); Df_A_AAH_IV, *Dictyostelium fasciculatum* endoribonuclease L-PSP domain-containing protein (EGG21287); Xl_A_AAH_IV, *Xenopus laevis* ATP binding domain 4 (NP 001085655); Hs_A_AAH_IV, Human ATP binding domain containing protein 4 (NP 542381); Mm_A_AAH_IV, mouse ATP binding domain containing protein 4 (NP 079951); Hs_UK114, human ribonuclease UK114/p14.5/L-PSP (NP 005827); Mm_UK114, mouse UK114/p14.5/L-PSP (NP 0032313). Note that the last two sequences appear twice in the alignment so that the sequence relationships to each of the YjgF-YER057c-UK114 related domains in the non-mammalian proteins can be shown. *, conserved residues shown to be important for trimerisation and ligand binding [63], [66].
(TIF)

Figure S9 Conservation of the *DPH7* gene product, Dph7. (A) Representation of Dph7 showing the location of the conserved WD40 domains. (B) The Dph7 amino acid sequence was aligned using Clustal with representative examples of putative orthologs from other organisms (identified by PSI-BLAST). Sequences are as follows (database accession numbers in parentheses): *DPH7*, *S. cerevisiae* Dph7/Ybr246w Sp_WD85, *Schizosaccharomyces pombe* WD repeat protein (CAA21429); At_WD85, *Arabidopsis thaliana* WD40 domain-containing protein (NP 201106); Dd_WD85, *Dictyostelium discoideum* WD40 repeat-containing protein (XP 646601); Xt_WD85, *Xenopus tropicalis* WD repeat-containing protein 85-like (XP 002942023); Hs_WD85, Human WD repeat-containing protein 85 (NP 620133); Mm_WD85, mouse unnamed protein (BAE 32074).
(TIF)

Table S1 SGA-based Excel spreadsheet extracted from the DRYGIN database for comprehensive presentation of genetic interactions between query genes *DPH1*, *DPH2*, *DPH4*, *DPH5*, *DPH6/YLR143w* and *DPH7/YBR246w* and array ORFs totaling 3885 (*DPH1*, *DPH2*, *DPH6/YLR143w* and *DPH7/YBR246w*) and 4457 (*DPH4* and *DPH5*). Genetic interaction profiles among the six queries were ranked according to Pearson Correlation Coefficient determination (PCC). Correlation scores of the top ten interactors identified with each query gene identified a tightly clustered and highly robust, SGA-based *DPH* gene network (Figure 2C).
(XLSX)

Table S2 Strains used or generated for this study.
(DOCX)

Table S3 Plasmids used or constructed for this study.
(DOCX)

Table S4 Primers and oligonucleotides used for this study.
(DOCX)
