# Supplementary figures and images for: The Amidation Step of Diphthamide Biosynthesis in Yeast Requires DPH6, a Gene Identified through Mining the DPH1-DPH5 Interaction Network
Source: PLoS Genet. 2013 Feb 28;9(2):e1003334. doi: 10.1371/journal.pgen.1003334 (PMC3585130; doi:10.1371/journal.pgen.1003334)

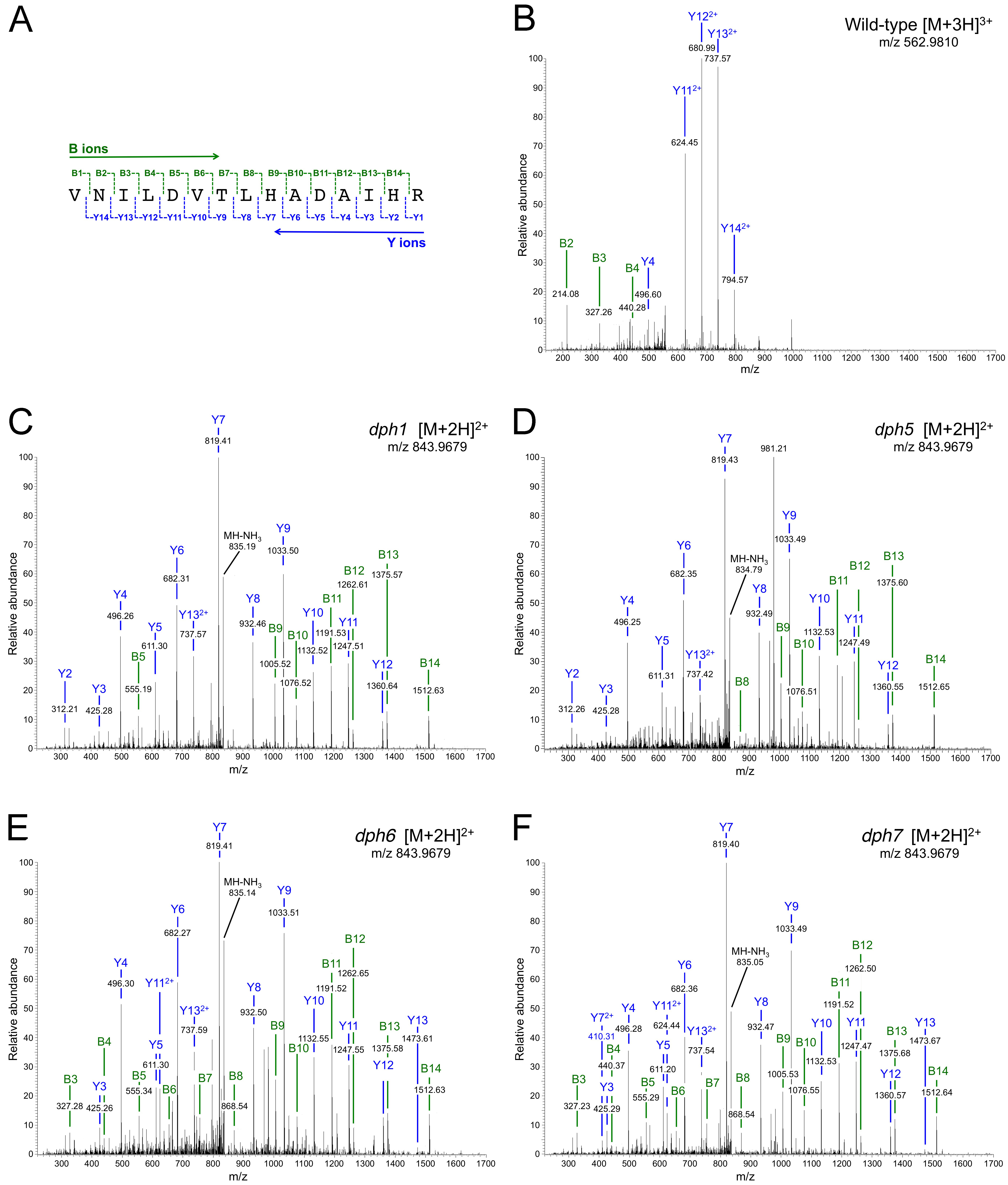

Supplement: Figure S1 — MS/MS spectra of unmodified eEF2 peptide 686-VNILDVTLHADAIHR-700 from wild-type and mutant yeast strains. (A) Cartoon showing how the B and Y ions seen in the MS/MS spectra map onto the tryptic peptide containing His-699. Y1 to Y13 and B14 ions contain His-699 and their m/z values are therefore informative regarding the modification state of His-699. (B–F) MS/MS spectra of unmodified peptide in eEF2 obtained from the indicated yeast strains: the parent ion m/z and charge state is indicated in each case. (TIF) [file pgen.1003334.s001.tif]

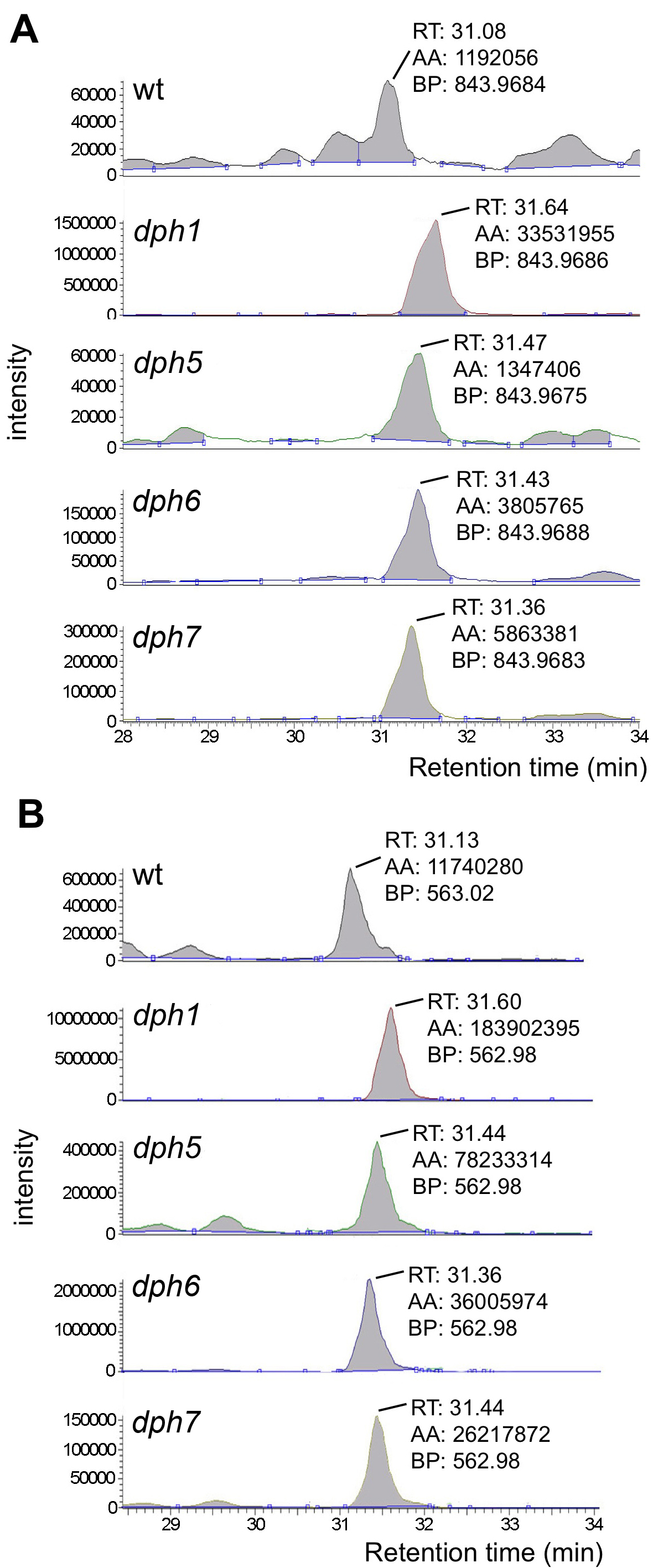

Supplement: Figure S2 — Extracted ion chromatograms of unmodified EF2 peptide 686-VNILDVTLHADAIHR-700. In (A), peaks corresponding to doubly-charged ions (m/z unmodified peptide 843.97, extracted mass range 843.8–844.0) are shown while triply-charged ions (m/z unmodified peptide 562.98, extracted mass range 562.5–563.2) are shown in (B). The yeast strain to which each chromatogram pertains is indicated. Note that in (B) an intensity of 580,000 corresponding to unmodified peptide with m/z 562.98 was not resolved from a different, more abundant ion with m/z 563.02 in the wt sample. Peak annotations are as follows: RT, retention time; AA, peak area; BP, parent ion m/z. (TIF) [file pgen.1003334.s002.tif]

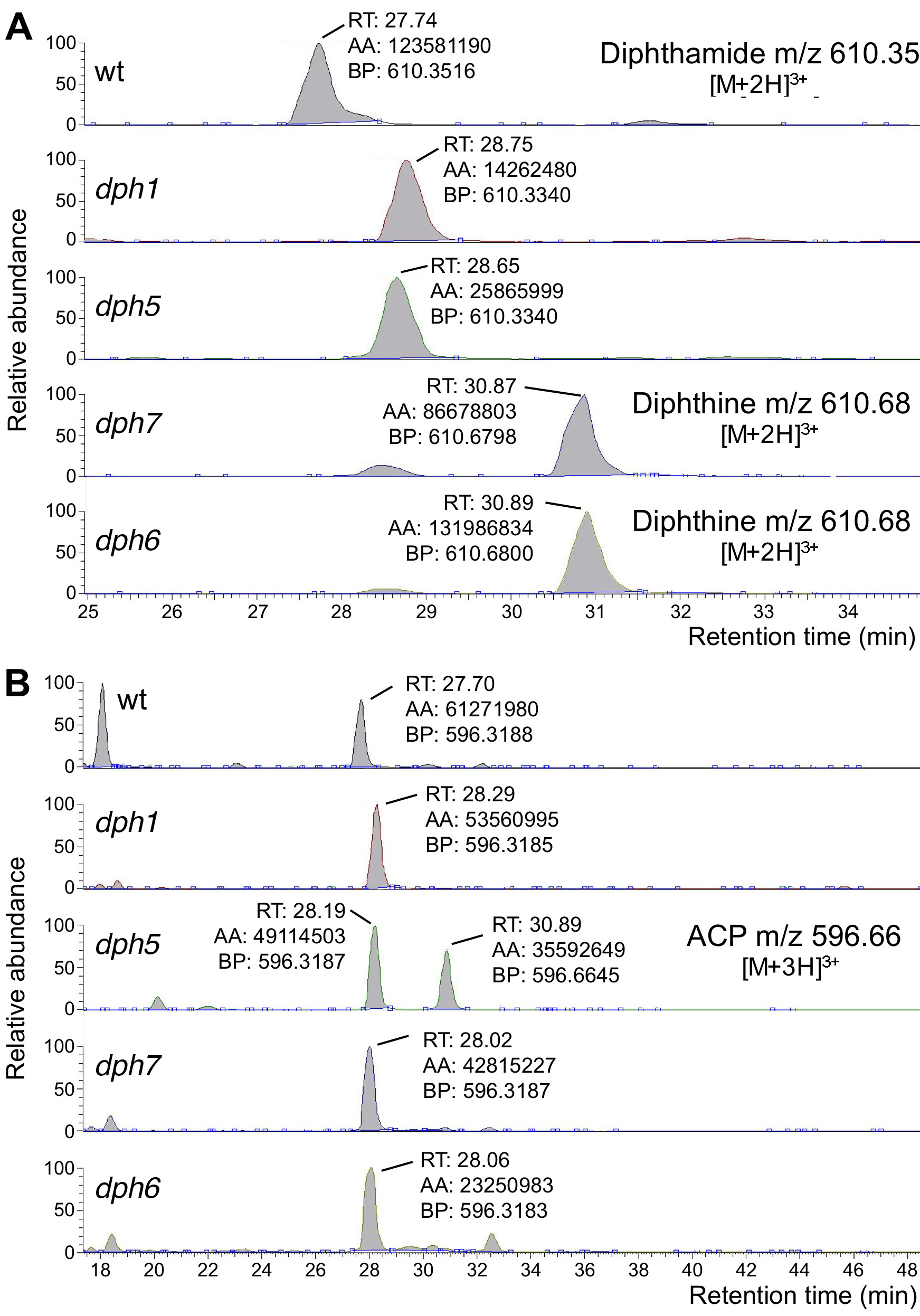

Supplement: Figure S3 — Extracted ion chromatograms of modified eEF2 peptide 686-VNILDVTLHADAIHR-700. (A) Peaks corresponding to triply-charged ions (m/z diphthine-modified peptide 610.68, m/z diphthamide-modified peptide 610.35, extracted masses 610.2–610.9). (B) Triply-charged ions (m/z ACP-modified peptide 596.66, extracted masses 596.2–596.8). Peak annotations are as follows: RT, retention time; AA, peak area; BP, parent ion m/z. (TIF) [file pgen.1003334.s003.tif]

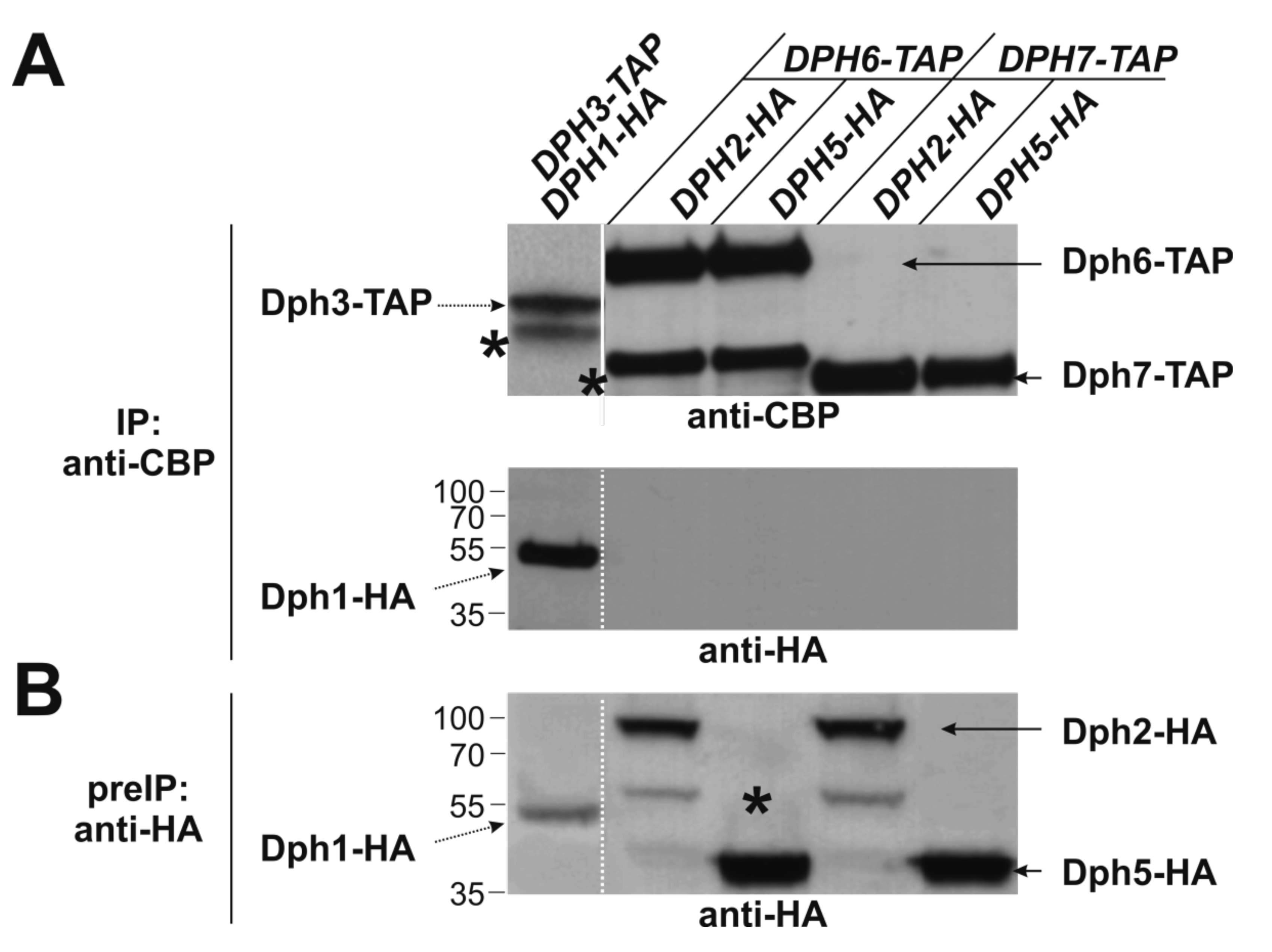

Supplement: Figure S4 — Failure to detect interaction by TAP-based co-immune precipitation between Dph6 or Dph7 and either Dph2 or diphthine synthase Dph5, factors integral to the first two steps of diphthamide synthesis. Co-immune precipitations were performed using magnetic beads (Dynabeads, Invitrogen) coupled to anti-CBP antibodies (Santa Cruz Biotechnology) specific for the calmodulin binding peptide (CBP) of the TAP-tag. The indicated strains expressed DPH6-TAP or DPH7-TAP in conjunction with HA-tagged versions of either DPH2 or DPH5. A strain co-expressing respectively, HA- and TAP-tagged variants of Dph1 and Dph3, step 1 pathway players previously shown to associate with one another [6], [20] served as a positive internal control for interaction. The presence of the respective proteins within the immune precipitates (IP) was assessed using anti-HA and anti-CBP Western blots (A) or anti-HA immune blots on total protein extracts obtained prior to the IP protocol (preIP). (B). Asterisks indicate breakdown products of Dph2-HA, Dph3-TAPand Dph6-TAP. (TIF) [file pgen.1003334.s004.tif]

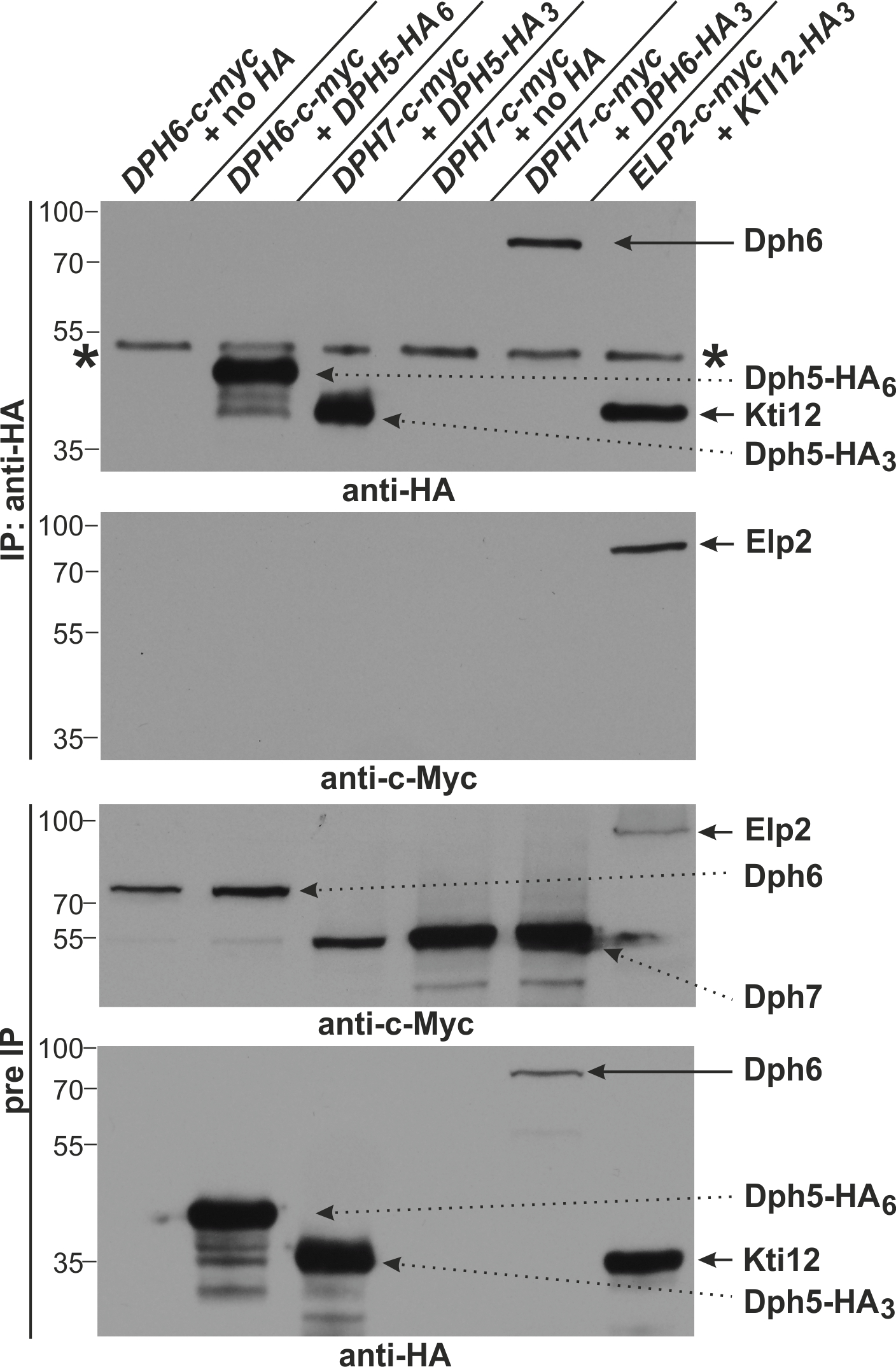

Supplement: Figure S5 — Failure to detect Dph6-Dph7 interaction by co-immune precipitation. Co-immune precipitations using the anti-HA-antibody were performed with the indicated strains expressing DPH6-c-myc or DPH7-c-myc on their own or in parallel with HA-tagged versions of DPH5 or DPH6, respectively. A strain co-producing c-Myc- and HA- and tagged versions of the Elp2 subunit (ELP2-c-myc) of the Elongator complex, and Kti12 (KTI12-HA), a protein known to interact with Elp2 [84], was used as internal positive control. The presence of the respective proteins was assessed in individual anti-c-Myc and anti-HA Western blots both in the IPs (top two panels) and crude extracts (pre IP; bottom two panels). The asterisk denotes an unspecific band that originates from the anti-HA-antibody present in the IPs. (TIF) [file pgen.1003334.s005.tif]

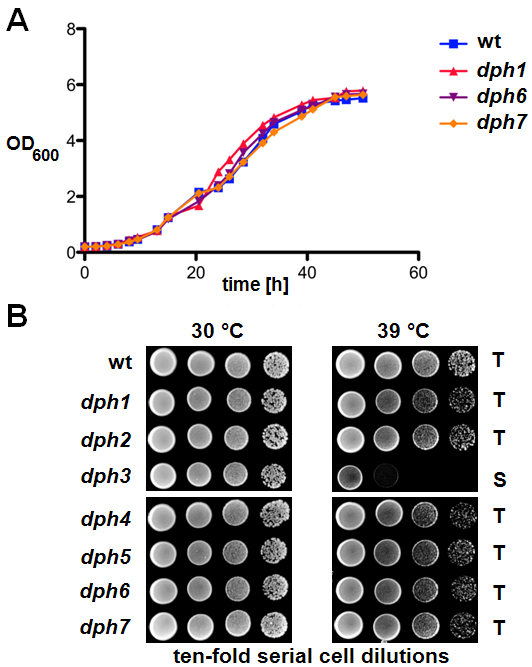

Supplement: Figure S6 — Lack of effect of dph1-dph7 gene knockouts on growth performance and viability. (A) The wild-type parental strain and diphthamide deficient mutants dph1, dph6 and dph7 were grown in YNB minimal media supplemented with His, Met, Ura, Leu to cover the auxotrophic markers (Table S2) under standard laboratory conditions over a period of 50 h. OD600 was monitored at 2 h intervals. (B) To address a potential temperature sensitive phenotype, ten-fold serial cell dilutions of the indicated strains were spotted on YPD plates and grown at 30°C or 39°C. Note that only the dph3/kti11 mutant, which affects additional biosynthetic pathways [6], [85] apart from diphthamide biosynthesis [13] shows temperature sensitivity (S) (S) while the other dph mutants tolerate high temperatures (T). (TIF) [file pgen.1003334.s006.tif]

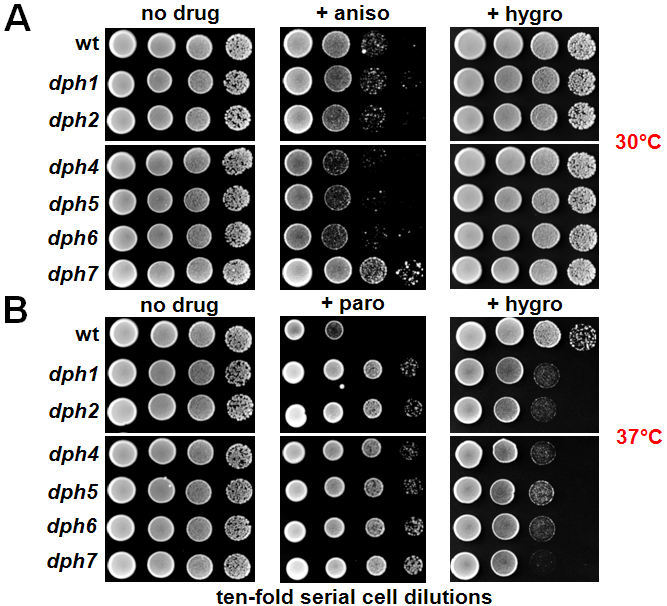

Supplement: Figure S7 — Altered growth performance of dph1-dph7 mutants in response to translation elongation indicator drugs under standard or elevated cultivation temperatures. Ten-fold serial cell dilutions of wild-type parental strain as well as diphthamide mutants dph1-dph7 were replica spotted on YPD plates without (control) and supplemented with hygromycin (20 µg/ml), anisomycin (20 µg/ml) or paromomycin (1.5 mg/ml) and grown at 30°C (A) or 37°C (B). Reduced or improved performance of the dph mutants relative to wild-type behavior reflects respectively, enhanced sensitivity or improved tolerance towards the drug in question respectively. (TIF) [file pgen.1003334.s007.tif]

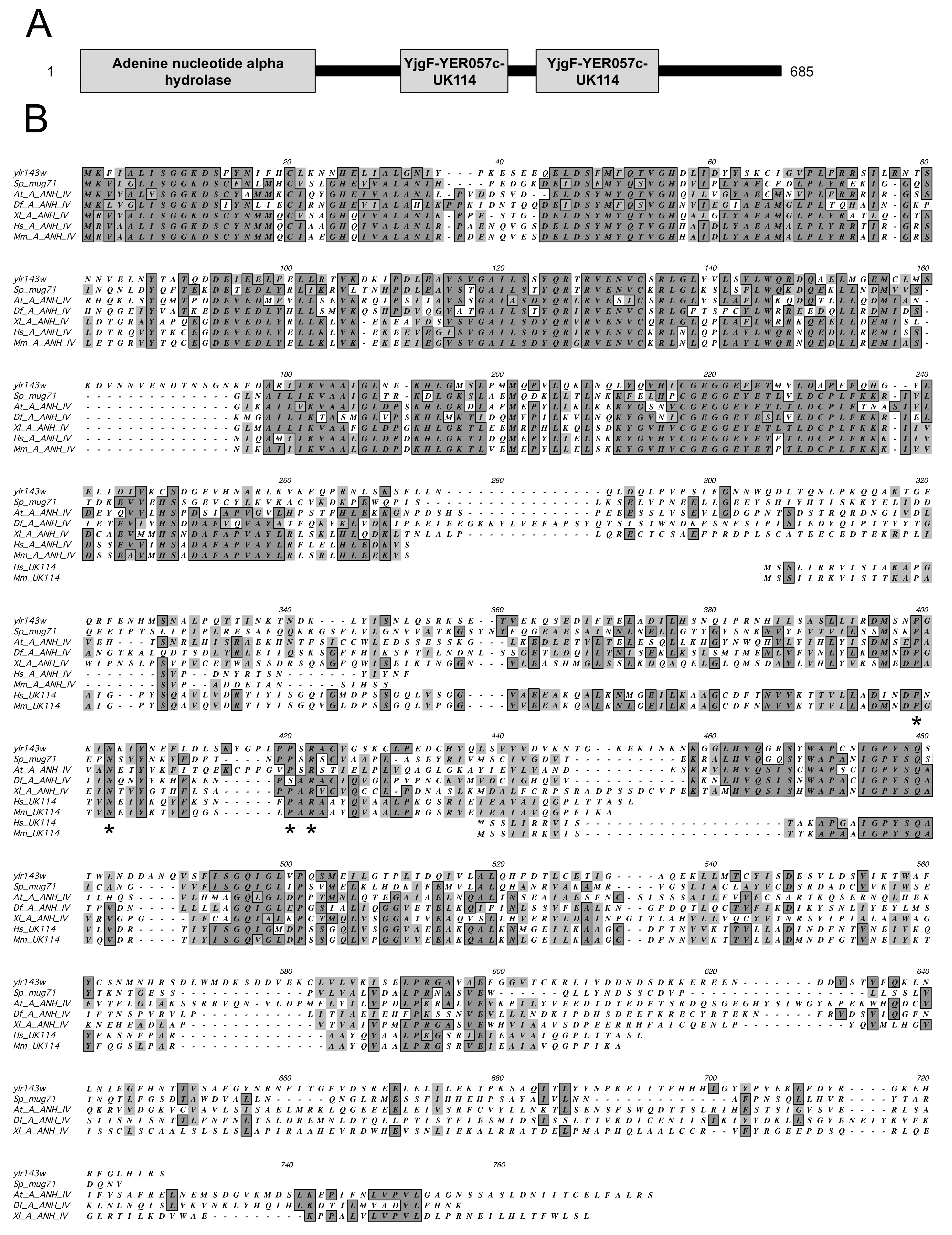

Supplement: Figure S8 — Conservation of the DPH6 gene product, Dph6. (A) Representation of Dph6 indicating the conserved adenine nucleotide alpha hydrolase (cd1994) and YjgF-YER057c-UK114 related (cd06155, cd06166) domains discussed in the main text. (B) The Dph6 amino acid sequence was aligned using Clustal with representative examples of putative orthologs from other organisms (identified by PSI-BLAST). Sequences are as follows (database accession numbers in parentheses): DPH6, S. cerevisiae Dph6/Ylr143w; Sp_mug71, Schizosaccharomyces pombe (NP 595310); At_A_AAH_IV, Arabidopsis thaliana endoribonuclease (NP 187098); Df_A_AAH_IV, Dictyostelium fasciculatum endoribonuclease L-PSP domain-containing protein (EGG21287); Xl_A_AAH_IV, Xenopus laevis ATP binding domain 4 (NP 001085655); Hs_A_AAH_IV, Human ATP binding domain containing protein 4 (NP 542381); Mm_A_AAH_IV, mouse ATP binding domain containing protein 4 (NP 079951); Hs_UK114, human ribonuclease UK114/p14.5/L-PSP (NP 005827); Mm_UK114, mouse UK114/p14.5/L-PSP (NP 0032313). Note that the last two sequences appear twice in the alignment so that the sequence relationships to each of the YjgF-YER057c-UK114 related domains in the non-mammalian proteins can be shown. *, conserved residues shown to be important for trimerisation and ligand binding [63], [66]. (TIF) [file pgen.1003334.s008.tif]

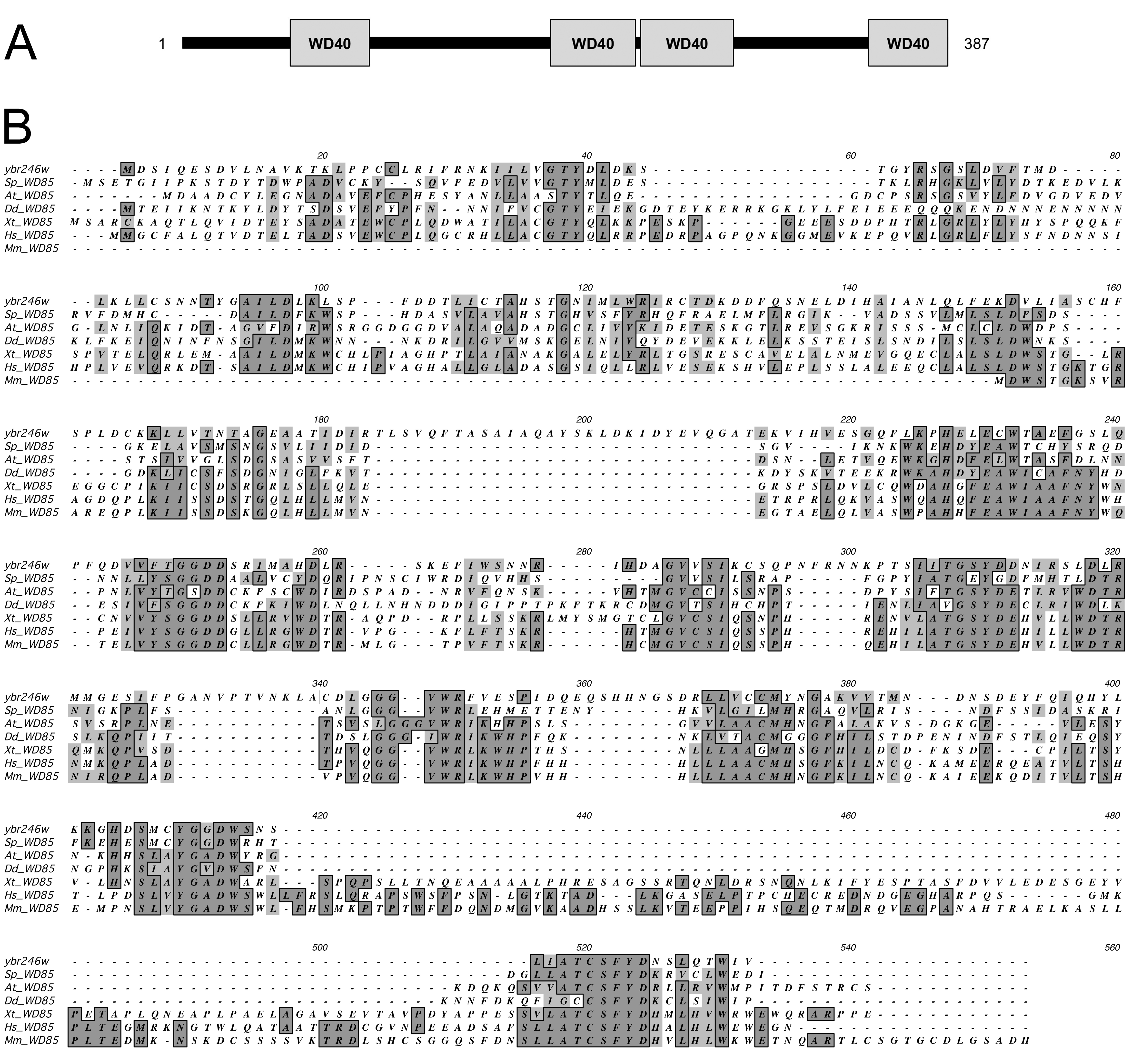

Supplement: Figure S9 — Conservation of the DPH7 gene product, Dph7. (A) Representation of Dph7 showing the location of the conserved WD40 domains. (B) The Dph7 amino acid sequence was aligned using Clustal with representative examples of putative orthologs from other organisms (identified by PSI-BLAST). Sequences are as follows (database accession numbers in parentheses): DPH7, S. cerevisiae Dph7/Ybr246w Sp_WD85, Schizosaccharomyces pombe WD repeat protein (CAA21429); At_WD85, Arabidopsis thaliana WD40 domain-containing protein (NP 201106); Dd_WD85, Dictyostelium discoideum WD40 repeat-containing protein (XP 646601); Xt_WD85, Xenopus tropicalis WD repeat-containing protein 85-like (XP 002942023); Hs_WD85, Human WD repeat-containing protein 85 (NP 620133); Mm_WD85, mouse unnamed protein (BAE 32074). (TIF) [file pgen.1003334.s009.tif]
