# Supplementary material for: The Amidation Step of Diphthamide Biosynthesis in Yeast Requires DPH6, a Gene Identified through Mining the DPH1-DPH5 Interaction Network
Source: PLoS Genet. 2013 Feb 28;9(2):e1003334. doi: 10.1371/journal.pgen.1003334 (PMC3585130; doi:10.1371/journal.pgen.1003334)
Supplement: Table S2 — Strains used or generated for this study. (DOCX) [file pgen.1003334.s011.docx]

**Table S2.** Strains used or generated for this study.

| Strain | Description | Fig(s) | Ref | |  |
| --- | --- | --- | --- | --- | --- |
|  |  |  |  | |  |
| BY4741 | *MAT***a** *his3*Δ*1 leu2*Δ*0 met15*Δ*0 ura3*Δ*0* | 3,6,S6-7 | Euroscarf | |  |
| Y02262 | BY4741 but *dph1*Δ*::KanMX4* | 3,6,S6-7 | Euroscarf | |  |
| Y05041 | BY4741 but *dph2*Δ*::KanMX4* | 3,6, S6-7 | Euroscarf | |  |
| CBY12 | BY4741 but *dph3*Δ*::SpHIS5* | 3,6, S6 | 1 | |  |
| Y06909 | BY4741 but *dph4*Δ*::KanMX4* | 3,6, S6-7 | Euroscarf | |  |
| Y04121 | BY4741 but *dph5*Δ*::KanMX4* | 3,6, S6-7 | Euroscarf | |  |
| Y04100 | BY4741 but *dph6/ylr143w*Δ*::kanMX4* | 3,6, S6-7 | Euroscarf | |  |
| Y03386 | BY4741 but *dph7*/*ybr246w*Δ*::kanMX4* | 3,6, S6-7 | Euroscarf | |  |
| MKK | *eft1*Δ*::LEU2 eft2*Δ*::HIS3* + p*EFT2* ([H699] *CEN URA3* | 3 | 2 | |  |
| MKK-I | *eft1*Δ*::LEU2 eft2*Δ*::TRP1* + p*EFT2* ([H699I] *CEN TRP1* | 3 | 2 | |  |
| MKK-N | *eft1*Δ*::LEU2 eft2*Δ*::HIS3* + p*EFT2* ([H699N] *CEN TRP1* | 3 | 2 | |  |
| SUY3 | BY4741 but *eft2*Δ*::KanMX4* + pTKB612 | S1-3.4-5 | This study | |  |
| SUY5 | SUY3 but *dph1*Δ*::* | S1-3 | This study | |  |
| SUY6 | SUY3 but *dph5*Δ*::KlURA3* | S1-3,4 | This study | |  |
| SUY4 | SUY3 but *dph6*Δ*::KlURA3* | S1-3,4 | This study | |  |
| SUY14 | SUY3 but *dph7*Δ*::KlURA3* | S1-3,4 | This study | |  |
| *DPH6-TAP* | YSC1178 but *DPH7-TAP::His3MX6* | - | This study | |  |
| *DPH7-TAP* | YSC1178 but *DPH7-TAP::His3MX6* | - | This study | |  |
| SUY36 | *DPH6-TAP* but *DPH2-HA::KanMX6* | S4 | This study | |  |
| SUY37 | *DPH6-TAP* but *DPH5-HA::KanMX6* | S4 | This study | |  |
| SUY38 | *DPH7-TAP* but *DPH2-HA::KanMX6* | S4 | This study | |  |
| SUY39 | *DPH7-TAP* but *DPH5-HA::KanMX6* | S4 | This study | |  |
| 1679-08A | *MAT***a** *leu2*Δ*1*  *trp1*Δ*63* *ura3-52 his3*Δ*200* *GAL* | - | Euroscarf | |  |
| LF11-TAP | 1679-08A, but *KTI11-TAP::KlTRP1* | S4 | 3 | |  |
| FFY2/4-dt | 1679-08A but *ELP2-(c-myc)_3_*::*SpHIS5 KTI12-(HA)_6_*::*KlTRP1* | S5 | 4 | |  |
| SUY43 | SUY3 but *DPH5-(HA)_6_::HIS3MX6* | 5 | This study | |  |
| CBKY3 | SUY3 but *DPH5-(HA)_6_::HIS3MX6 dph1*Δ*::URA3* | 5 | This study | |  |
| CBKY2 | SUY3 but *DPH5-(HA)_6_::HIS3MX6 dph6*Δ*::URA3* | 5 | This study | |  |
| CBKY1 | SUY3 but *DPH5-(HA)_6_::HIS3MX6 dph7*Δ*::URA3* | 5 | This study | |  |
| CBKY6 | SUY3 but *DPH6-(HA)_3_::KanMX6* | 5 | This study | |  |
| CBKY12 | SUY3 but *DPH6-(HA)_3_::KanMX6 dph7*Δ*::HIS3* | 5 | This study | |  |
| CBKY7 | BY4741 but *DPH6-(c-myc)_3_::KanMX6* | S5 | This study | |  |
| CBKY11 | CBKY7 but *DPH5-(HA)_6_::HIS3MX6* | S5 | This study | |  |
| CBKY8 | BY4741 but *DPH7-(c-myc)_9_::HIS3MX6* | S5 | This study | |  |
| CBKY9 | CBKY8 but *DPH5-(HA)_3_::KanMX6* | S5 | This study | |  |
|  |  |  | |  | |
|  |  |  | |  | |

1. Bär C, Zabel R, Liu S, Stark MJ, Schaffrath R (2008) A versatile partner of eukaryotic protein complexes that is involved in multiple biological processes: Kti11/Dph3. Mol Microbiol 69: 1221-1233

2. Kimata Y, Kohno K (1994) Elongation factor 2 mutants deficient in diphthamide formation show temperature-sensitive cell growth. he J Biol Chem 269: 13497-13501.

3. Fichtner L, Jablonowski D, Schierhorn A, Kitamoto HK, Stark MJ, et al. (2003) Elongator's toxin-target (TOT) function is nuclear localization sequence dependent and suppressed by post-translational modification. Mol Microbiol 49: 1297-1307.

4. Fichtner L, Frohloff F, Bürkner K, Larsen M, Breunig KD, Schaffrath R (2002) Molecular analysis of *KTI12*/*TOT4*, a *Saccharomyces cerevisiae* gene required for *Kluyveromyces lactis* zymocin action. Mol Microbiol 43: 783-791.
