# Supplementary material for: The Amidation Step of Diphthamide Biosynthesis in Yeast Requires DPH6, a Gene Identified through Mining the DPH1-DPH5 Interaction Network
Source: PLoS Genet. 2013 Feb 28;9(2):e1003334. doi: 10.1371/journal.pgen.1003334 (PMC3585130; doi:10.1371/journal.pgen.1003334)
Supplement: Table S3 — Plasmids used or constructed for this study. (DOCX) [file pgen.1003334.s012.docx]

**Table S3.** Plasmids used or constructed for this study.

| Strain | | Description | Figs | | Ref | |  |  |  |  |
| --- | --- | --- | --- | --- | --- | --- | --- | --- | --- | --- |
|  | | |  |  | | | | |  |  |
| pLMY101 | | YEp p*GAL1*-*DTA* (DT ADP ribosylase domain) *URA3* *2μ* | | - | | 1 | | | | |
| pSU6 | | YCplac111 carrying *DPH6* and flanking regions as an *Eco*RI-*Bam*HI fragment; *LEU2* *CEN-ARS* | | 7 | | This study | | | | |
| pSU7 | | YEplac181 carrying *DPH6* and flanking regions as an *Eco*RI-*Bam*HI fragment; *LEU2* *2μ* | | 7 | | This study | | | | |
| pMS61/62 | | pSU6 carrying *DPH6* G216N, E220A double point mutation | | 7 | | This study | | | | |
| pMS67/68 | | pSU6 but with *DPH6* residues 335 onwards replaced by *myc_3_* | | 7 | | This study | | | | |
| pMS72 | | pSU7 but with in-frame deletion of *DPH6* codons 347-471 | | 7 | | This study | | | | |
| pSU8/9 | | YCp p415/46-*GALS* + *DTA* *BamH1*insert of pLMY101 | | 3 | | This study | | | | |
| p415/16-GALS | | YCp pRS415/16 + p*GALS* prom. *LEU2/URA3* *CEN-ARS* | | - | | 2 | | | | |
| pTKB612 | | YCp p*TEF5*-*EFT2-(His)_6_ CEN-ARS LEU2* | | S1-3,4-5 | | 3 | | | | |
| pJD204.0 | | YCp pRS316: *lacZ* no frameshift [FS] *CEN-ARS URA3* | | 6 | | 4 | | | | |
| pJD204.-1 | | YCp pRS316: *lacZ* -1 frameshift [FS] *CEN-ARS URA3* | | 6 | | 4 | | | | |
| pJD204.+1 | | YCp pRS316: *lacZ* +1 frameshift [FS] *CEN-ARS URA3* | | 6 | | 4 | | | | |
| pGAL-*DPH5* | | YEp BG1805: p*GAL1*-*DPH5-HA-(His)_6_ URA3* *2μ* | | 6 | | Open Biosystems | | | | |
| pGAL-*DPH1* | | YEp BG1805: p*GAL1*-*DPH1-HA-(His)_6_ URA3* *2μ* | | S4 | | Open Biosystems | | | | |
|  | | |  |  |  | |  |  |  |  |

1. Mattheakis LC, Shen WH, Collier RJ (1992) *DPH5*, a methyltransferase gene required for diphthamide biosynthesis in *Saccharomyces cerevisiae*. Mol Cell Biol 12: 4026-4037.

2. Mumberg D, Muller R, Funk M (1994) Regulatable promoters of *Saccharomyces cerevisiae*: comparison of transcriptional activity and their use for heterologous expression. Nucleic Acids Res 22: 5767-5768.

3. Jørgensen R, Carr-Schmid A, Ortiz PA, Kinzy TG, Andersen GR (2002) Purification and crystallization of the yeast elongation factor eEF2. Acta Crystallogr D Biol Crystallogr 58: 712-715.

4. Ortiz PA, Ulloque R, Kihara GK, Zheng H, Kinzy TG (2006) Translation elongation factor 2 anticodon mimicry domain mutants affect fidelity and diphtheria toxin resistance. J Biol Chem 281: 32639-32648.
