# Supplementary material for: The Amidation Step of Diphthamide Biosynthesis in Yeast Requires DPH6, a Gene Identified through Mining the DPH1-DPH5 Interaction Network
Source: PLoS Genet. 2013 Feb 28;9(2):e1003334. doi: 10.1371/journal.pgen.1003334 (PMC3585130; doi:10.1371/journal.pgen.1003334)
Supplement: Table S4 — Primers and oligonucleotides used for this study. (DOCX) [file pgen.1003334.s013.docx]

**Table S4.** Primers and oligonucleotides used for this study.

| Name | | Sequence (5’🡪3’) | | Use | |  |
| --- | --- | --- | --- | --- | --- | --- |
|  | | |  | |  | |
| fw*DPH*6-ko | | CAATAAGTCAGTATCATGAAGTTTATAGCATTAATATCAGGTGGGAAGCGACGGCCAGTGAATTCCCGG | | *DPH*6 gene deletion | |  |
| rv*DPH*6-ko | | CATTTGGAGTTAGGAACGAATATGCAACCCAAAGCGGTGTTCTTTACCAGCTTGGCTGCAGGTCGACGG | | *DPH*6 gene deletion | |  |
| fw*DPH6* | | CCAAAAAGAGTAGGCCTATGAGAGG | | *DPH*6 deletion diagnosis | |  |
| rv*DPH6* | | CTTGGTCTTTAGCTTATTCAGGTGC | | *DPH*6 deletion diagnosis | |  |
| fw*DPH*7-ko | | CTACATCCACCTCTAGCTGGTTTTTGCATAGCTATACATATGGACCGACGGCCAGTGAATTCCCGG | | *DPH7* gene deletion | |  |
| rv*DPH*7-ko | | CTAAACTATCCATGTTTGCAAGGAATTATCATAAAATGAGCATGTTGAGCTTGGCTGCAGGTCGACGG | | *DPH7* gene deletion | |  |
| fw*DPH7* | | GCAGAGACACCAACCAGTTGACACC | | *DPH*7 deletion diagnosis | |  |
| rv*DPH7* | | CTATTGGCAGGAGCCAGCAGGGAG | | *DPH*7 deletion diagnosis | |  |
| S2-*DPH1* | | GAATATGATACTAACTATTTATACATATGTAACAGGAAGACAAGTGACAACAAAAACTATTTAAAATCGATGAATTCGAGCTCG | | *DPH1* epitope tagging | |  |
| S3-*DPH1* | | ATCCAATGGATTATTACGAAGCTAAAGGATACGGGCGTGGGGAAACTCCGAAACATGCGATTGAACGTACGCTGCAGGTCGAC | | *DPH1* epitope tagging | |  |
| S2-*DPH2* | | TAAATAGTTTATTAGTTAAAATCTTGGATTTAAATAGAGAAGTCGAGGGAAACAAATTATAAGAGATCGATGAATTCGAGCTCG | | *DPH2* epitope tagging | |  |
| S3-*DPH2* | | GTATTTCCGGTGTCGCACGTGGTTATGGATTTGATCGCGAAGACGCTATGAAAAAGGAAAACAAACGTACGCTGCAGGTCGAC | | *DPH2* epitope tagging | |  |
| S2-*DPH5* | | GGCCCGATTCGTTTGGGATCGAATTGTTACCCGACTGAAAGGATCGATGAATTCGAGCTCG | | *DPH5* epitope tagging | |  |
| S3-*DPH5* | | CGGCATGGGTCCCACCCACAGAAGACGACAGCGACGAGCGTACGCTGCAGGTCGAC | | *DPH5* epitope tagging | |  |
| S2-*DPH6* | | GCGTATATCTATTAAGTTTATAAAATATAAGGCCTACATTTGGAGATCGATGAATTCGAGCTCG | | *DPH6* epitope tagging | |  |
| S3-*DPH6* | | GATTACCGTGGTAAAGAACACCGCTTTGGGTTGCATATTCGTTCCCGTACGCTGCAGGTCGAC | | *DPH6* epitope tagging | |  |
| S2-*DPH7* | | CCTATATATTAGCCTATATATTAGCCTATATATTAGTCCATATATTGCAGACTGAATTTATCGATGAATTCGAGCTCG | | *DPH7* epitope tagging | |  |
| S3-*DPH7* | | GTCAAATTCTTTGATCGCAACATGCTCATTTTATGATAATTCCTTGCAAACATGGATAGTTCGTACGCTGCAGGTCGAC | | *DPH6* epitope tagging | |  |
| YLR143w-Fw-*Eco*R1 | | CGAATTCGATTATCTTTTGAGAAGATTGCAAG | | *DPH6* cloning | |  |
| YLR143w-Rv-*Bam*H1 | | CGGATCCCGTGGACAGCGTATATCTATTAAG | | *DPH6* cloning | |  |
|  | | |  | |  | |
